# Dynamical Localization of DivL and PleC in the Asymmetric Division Cycle of *Caulobacter crescentus*: A Theoretical Investigation of Alternative Models

**DOI:** 10.1371/journal.pcbi.1004348

**Published:** 2015-07-17

**Authors:** Kartik Subramanian, Mark R. Paul, John J. Tyson

**Affiliations:** 1 Graduate Program in Genetics, Bioinformatics and Computational Biology, Virginia Polytechnic Institute and State University, Blacksburg, Virginia, United States of America; 2 Department of Mechanical Engineering, Virginia Polytechnic Institute and State University, Blacksburg, Virginia, United States of America; 3 Department of Biological Sciences, Virginia Polytechnic Institute and State University, Blacksburg, Virginia, United States of America; 4 Virginia Bioinformatics Institute, Virginia Polytechnic Institute and State University, Blacksburg, Virginia, United States of America; ETH Zurich, SWITZERLAND

## Abstract

Cell-fate asymmetry in the predivisional cell of *Caulobacter crescentus* requires that the regulatory protein DivL localizes to the new pole of the cell where it up-regulates CckA kinase, resulting in a gradient of CtrA~P across the cell. In the preceding stage of the cell cycle (the “stalked” cell), DivL is localized uniformly along the cell membrane and maintained in an inactive form by DivK~P. It is unclear how DivL overcomes inhibition by DivK~P in the predivisional cell simply by changing its location to the new pole. It has been suggested that co-localization of DivL with PleC phosphatase at the new pole is essential to DivL’s activity there. However, there are contrasting views on whether the bifunctional enzyme, PleC, acts as a kinase or phosphatase at the new pole. To explore these ambiguities, we formulated a mathematical model of the spatiotemporal distributions of DivL, PleC and associated proteins (DivJ, DivK, CckA, and CtrA) during the asymmetric division cycle of a *Caulobacter* cell. By varying localization profiles of DivL and PleC in our model, we show how the physiologically observed spatial distributions of these proteins are essential for the transition from a stalked cell to a predivisional cell. Our simulations suggest that PleC is a kinase in predivisional cells, and that, by sequestering DivK~P, the kinase form of PleC enables DivL to be reactivated at the new pole. Hence, co-localization of PleC kinase and DivL is essential to establishing cellular asymmetry. Our simulations reproduce the experimentally observed spatial distribution and phosphorylation status of CtrA in wild-type and mutant cells. Based on the model, we explore novel combinations of mutant alleles, making predictions that can be tested experimentally.

## Author Summary

The aquatic bacterium, *Caulobacter crescentus*, divides asymmetrically into a non-motile “stalked” cell that stays at its place of birth, and a motile “swarmer” cell that disperses to a different locale. Prior to cell division, the cell passes through a “predivisional” stage, when it has a stalk at its “old” end and a flagellum at its “new” end. These spatiotemporal changes in morphology are driven, in part, by changes in subcellular localization of signaling proteins. To understand how the cell exploits protein localization to generate distinct cell fates, we formulated a mathematical model of the spatiotemporal dynamics of six regulatory proteins (DivJ, DivK, PleC, DivL, CckA and CtrA) during the *Caulobacter* cell cycle. Contrary to some suggestions, our model predicts that PleC functions as a kinase during the predivisional stage of the cell cycle. Further, we show that spatial separation of DivL and PleC kinase in the stalked stage is required for inactivation of DivL and for initiation of DNA synthesis. Later, co-localization of DivL and PleC kinase at the new pole of the cell restores DivL activity in the swarmer-half of the cell, resulting in the establishment of replicative asymmetry in the predivisional stage of the cell cycle.

## Introduction

The asymmetric localization of proteins is critical for cell and/or tissue development in eukaryotic systems as diverse as *S*. *cerevisiae* [1], *C*. *elegans* [2], *A*. *thaliana* [3], and *D*. *melanogaster* [4]. For years, spatial organization of cellular components was thought to be an exclusive feature of eukaryotes, but advances in microscopy and protein labeling over the past two decades have dispelled this notion [5]. The localization of cellular components—including lipids, DNA, RNA and proteins–is also an integral feature of prokaryotic cells; observed to play a role in the growth, function and survival of many bacteria, including *E*. *coli* [6], *B*. *subtilis* [7,8], *V*. *cholerae* [9], *S*. *flexnerii* [10,11]. However, with roughly 10% of its proteins having the potential to localize [12], *Caulobacter crescentus* serves as the model bacterium to study subcellular localization of proteins in prokaryotes. In *Caulobacter*, the non-uniform distribution of proteins is visibly manifested in the asymmetric division cycle that gives rise to two morphologically and functionally distinct daughter cells [13–15]. Furthermore, subcellular localization of macromolecules influences many physiological attributes of *Caulobacter* cells, such as growth [16,17], cell shape [18,19], morphogenesis [20], differentiation [21,22], stringent response [23,24], and cell division [25]. *Caulobacter* shares many regulatory genes with other species of alpha-proteobacteria, including species that are of importance to agriculture and medicine, such as the nitrogen-fixing *Sinorhizobium meliloti*, the plant pathogen *Agrobacterium tumefaciens*, and the mammalian pathogens *Rickettsia prowazekii* and *Brucella abortus* [26,27]. While mounting evidences show causal links between protein localization and cell function in these bacteria [20,28–34], the underlying molecular mechanisms that enable the cell to use subcellular protein gradients to achieve complex cellular behavior are not completely understood.

The bacterium *Caulobacter crescentus* undergoes asymmetric division to give rise to two non-identical daughter cells, called a stalked cell and a swarmer cell. The sessile and replication-competent stalked cell is anchored to the substratum, while the motile but replication-quiescent swarmer cell swims to a new locale, before shedding its flagellum and differentiating into a stalked cell. This dimorphism enables the bacterial population to disperse and survive in the low-nutrient, aquatic environments where *Caulobacter* is naturally found [15]. The precursor to asymmetric division is the predivisional cell, which is characterized by a stalk at one pole and nascent swarmer apparatus at the opposite pole. The swarmer, stalked and predivisional cells represent three distinct developmental stages that define the *Caulobacter* cell cycle. Progression through this cycle is dictated by the phosphorylation status of the master regulator CtrA, which serves as a transcription factor for nearly 100 genes [35]. In particular, by regulating expression of the hemimethyltransferase, CcrM, CtrA controls the methylation state of DNA in stalked and predivisional cells [36–40], and by binding to the origin of replication, the phosphorylated form of CtrA (CtrA~P) inhibits DNA replication in swarmer cells [41]. A gradient of CtrA phosphorylation is established in predivisional cells, with CtrA~P high in the swarmer end and low in the stalked end. As a result, one daughter cell inherits the phosphorylated form of CtrA, and the other daughter cell inherits the unphosphorylated form. Subsequently, different sets of proteins are expressed in the two cells, culminating in distinct swarmer and stalked cell morphologies.

**Fig 1:**
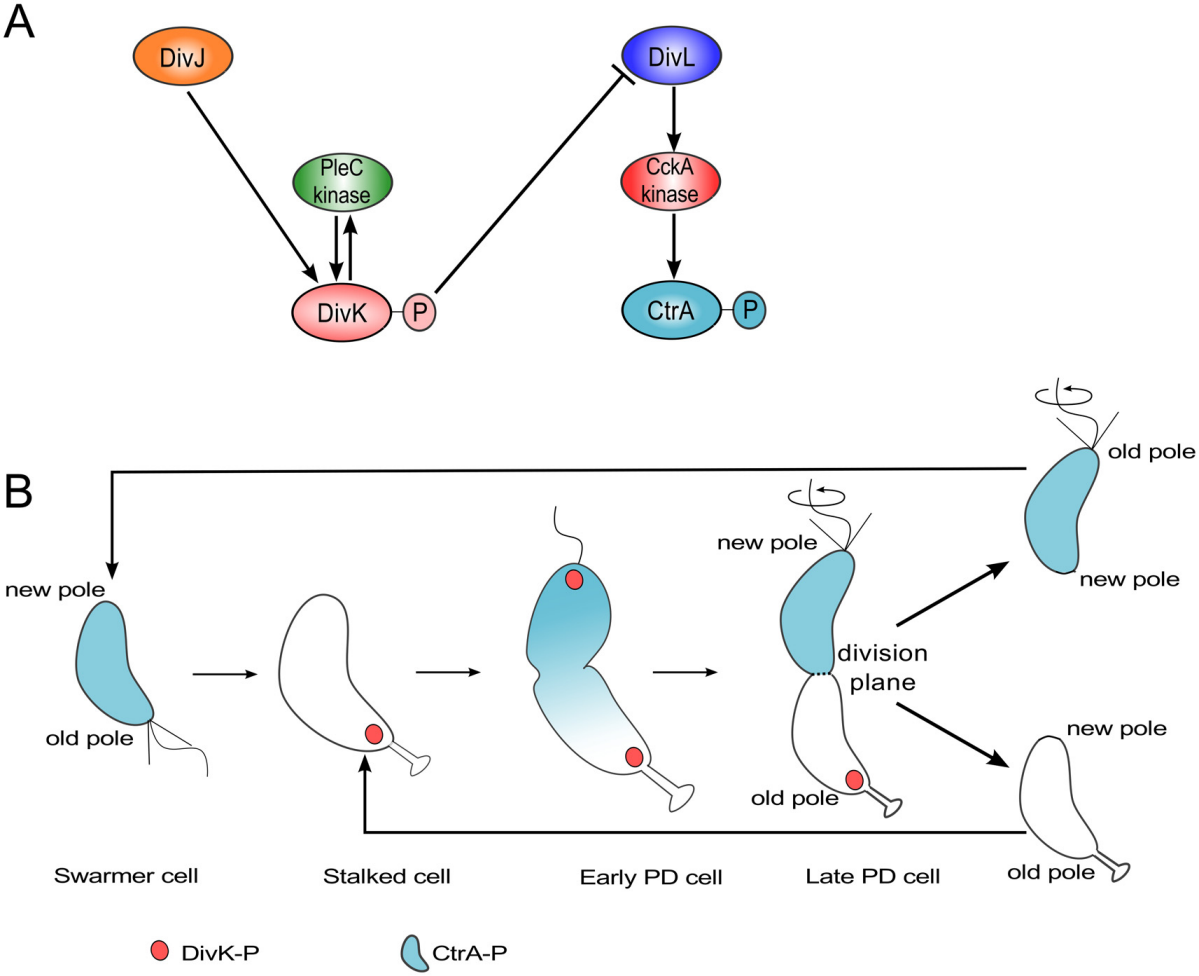
The regulation of DivK and CtrA phosphorylation during the cell division cycle of *Caulobacter crescentus*. **(A)** Influence diagram of two signal transduction pathways in *C*. *crescentus*. Barbed arrows indicate activation, while the blunt-headed line indicates inhibition. **(B)** Spatiotemporal distributions of DivK~P (red) and CtrA~P (light blue). PD = predivisional. Notice that both DivK and CtrA are phosphorylated in the PD cell.

CtrA and another response regulator, DivK, are at the termini of two phosphotransfer modules: DivJ-PleC-DivK and DivL-CckA-CtrA, see Fig 1A. PleC and CckA are bifunctional histidine-modifying enzymes that may act as either kinases or phosphatases for their respective response regulators, DivK and CtrA [42,43]. DivJ is the main kinase for phosphorylating DivK, while the role of DivL is to up-regulate CckA’s kinase activity [44]. DivL is a tyrosine kinase, but its kinase activity is not involved in the up-regulation of CckA [44]; how DivL promotes CckA activity is still unknown. An important step in the pathway is the inhibition of DivL by binding to DivK~P [45]. In a swarmer cell, DivJ is absent, PleC is a phosphatase, and DivK is unphosphorylated. Consequently, DivL is actively up-regulating CckA kinase activity [46], which in turn maintains CtrA in its phosphorylated form, thereby inhibiting DNA replication in the swarmer cell [41]. The introduction of DivJ during the swarmer-to-stalked transition enables the phosphorylation of DivK, triggering a pathway that culminates in the dephosphorylation of CtrA~P in stalked cells [47–49]. Therefore, at the molecular level, swarmer and stalked cells can be distinguished based on which response regulator—CtrA or DivK—is phosphorylated.

The schematic in Fig 1A suggests that DivL and CtrA cannot be concurrently phosphorylated during the cell cycle. Therefore, it was surprising to find that both response regulators are phosphorylated in predivisional cells (Fig 1B) [14,20,50]. While the level of DivK~P remains fairly constant in the stalked and predivisional stages [51], CtrA~P level changes sharply from lowest in the stalked cell stage to peak activity in the predivisional stage [43,52]. How does CtrA~P avoid DivK~P dependent inhibition only in predivisional cells? Recent experimental observations [45,46] have shown that the reason for phosphorylation of CtrA in the predivisional cell is the restoration of DivL activity (up-regulating CckA kinase) after it localizes to the new pole. In contrast, cells that are unable to localize DivL to the new pole fail to localize CckA or activate its kinase function. Taken together, these observations suggests that (a) phosphorylation of CtrA in predivisional cells is the result of failure of DivK~P to inhibit DivL, and (b) the location of DivL determines whether or not it can be inhibited by DivK~P. From the wiring diagram in Fig 1 it is not immediately apparent how the inhibitory interaction between DivK~P and DivL—which is required for the swarmer-to-stalked transition early in the cell cycle—is circumvented in the predivisional stage of the cell cycle simply because DivL relocates to the new pole.

One possible explanation for DivL reactivation is that, by localizing to the new pole, DivL comes in proximity to the bifunctional enzyme PleC, which may dephosphorylate and inactivate any incoming DivK~P, thus rendering it unable to inhibit DivL [45]. We shall refer to this informal model as ‘protection by dephosphorylation’. (We refer to verbal explanations of experimental findings as “informal models” to distinguish them from the mathematical models we explore in this paper.) The protection-by-dephosphorylation model assumes that new-pole PleC is functioning to dephosphorylate DivK~P. There are contrasting opinions as to whether PleC in a predivisional cell is a phosphatase [53] or a kinase [54–56]. Clearly, to understand how DivL relocation influences its reactivation requires knowledge of whether PleC is functioning primarily as a phosphatase or a kinase at the new pole of a predivisional cell. To the best of our knowledge, experimental methods to measure the functional status of PleC (or other bifunctional kinases) at a specific subcellular location have yet to be developed. Therefore, we have undertaken a mathematical modeling approach to address this question.

In earlier work, we described the temporal dynamics of the DivJ-PleC-DivK and DivL-CckA-CtrA network by a formal mathematical model (in terms of ordinary differential equations) that was consistent with the principles of thermodynamics, biochemical kinetics, and allostery [57]. In this paper, we extend our temporal model to include spatial aspects of protein localization (Methods and S1 and S2 Tables). Our objective is to use computational analysis to understand how the cell exploits dynamic localization of key enzymes, DivL and PleC, to regulate signal transduction and drive differentiation events.

It is important to note that, of the six proteins that are a part of this model, there is limited understanding of how DivJ [48,58], PleC [20,53,58–60], DivL [61,62], and CckA [52,61,62] are localized. Hence, we have refrained from a mechanistic description of the localization of these proteins in our current model. Instead, we enforce a set of rules for the localization of these four proteins. Given these rules, our reaction-diffusion equations determine the spatial location of DivK and CtrA and how the interact with DivJ, PleC, DivL and CckA. In particular, our equations determine whether PleC and CckA are functioning (in particular times and places) as kinases or phosphatases and, as a result, whether DivK and CtrA are phosphorylated or not.

We first investigated, for physiologically relevant values of diffusion constants, whether PleC functions as a kinase or phosphatase in the predivisional cell. Based on our simulation results, we favor the conclusion that PleC is a kinase in the predivisional cell, in contradiction to the protection-by-dephosphorylation model. As an alternative mechanism, we propose an ‘inhibitor-sequestration’ model, in which PleC kinase binds to and sequesters incoming DivK~P, thus rescuing DivL from inactivation. To test the feasibility of the inhibitor-sequestration idea (within the framework of our mathematical model), we alter the localization profiles of relevant proteins and compare the predictions of our model equations to experimentally observed localization of proteins in wild-type and mutant strains of *Caulobacter*. Finally, we make experimentally verifiable predictions regarding the distribution and phosphorylation profiles of CtrA and DivK.

## Methods

### Modeling framework

Our reaction-diffusion model (S1 Table) is based on the mechanism proposed in our earlier paper for regulation of bifunctional histidine kinases [57]. Readers should consult that paper for the rationale behind the reaction kinetics in S1 Table. The temporal model in [57] is expanded to include diffusion of proteins along the long axis of a *Caulobacter* cell and the localization of specific proteins to the poles of the cell during specific stages of the cell cycle. Because we are interested in protein patterns along the long axis and at the poles of the cell, it is sufficient to formulate the model for one spatial dimension. The governing partial differential equation (PDE) for a generic chemical species takes the form:

$$\frac{\partial C}{\partial t}=\;\text{Reaction terms}+\;D\cdot \;\frac{\partial^{2}C}{\partial x^{2}\;}$$

where *C*(*x*,*t*) is the concentration of species C at location *x* and time *t*. By discretizing the spatial dimension into *n* = 100 compartments of equal length *h* = *L*/*n*, where *L* is the total length of the *Caulobacter* cell, and using a central difference scheme to approximate the Laplacian operator, we convert each PDE into a set of ordinary differential equations (ODEs). In our notation, *C*_*i*_ is the concentration of species C in compartment *i* (1 ≤ *i* ≤ 100) where

$$\frac{d{\text{C}}_{i}}{dt}=\;\text{Reaction terms }+\;\;D\;\cdot \frac{{\text{C}}_{i+1}-2{\text{C}}_{i}+{\text{C}}_{i-1}}{h^{2}}$$

Because *Caulobacter* cells are elongating as a result of new cell wall material being added uniformly along the long axis [16], we assume that each compartment grows exponentially in time as

$$\frac{dh}{dt}=\;\;k_{\text{growth}}\;\cdot h$$

Since the molecules being investigated cannot diffuse across the cell wall, we implement no-flux boundary conditions at *x* = 0 and *x* = *L* by adding two additional compartments, *i* = 0 and *i* = 101, and insisting at every time step that *C*_0_ = *C*_1_ and *C*_101_ = *C*_100_. The reaction and diffusion rate constants for the wild type and mutant cells are provided in S2 and S3 Tables.

A complete understanding of the mechanisms governing localization of DivJ, PleC, DivL and CckA is lacking at this stage, and our model does not attempt to offer one. We enforce the localization of these four kinases based on experimentally observed distributions in wild-type and mutant cells [20,48,52,58,62]. We do this by defining rates of binding and unbinding of species C to docking proteins in compartment *i* as follows:

$$\frac{dC_{i}^{b}}{dt}=\;p_{i}\cdot \;k_{\text{binding}}\;\cdot C_{i}^{f}-k_{\text{unbinding}}\;\cdot C_{i}^{b}\;+\;\text{other terms}$$

where *p*_*i*_ is an indicator function that takes the value of 1 or 0, $C_{i}^{b}$ is the concentration of the localized form and $C_{i}^{f}$ is the concentration of the freely diffusing form of a generic protein in compartment *i* of the cell. The values of the indicator functions for DivJ, PleC, DivL and CckA are provided in S4 Table.

The full set of ODEs were simulated in MATLAB using the ode15s solver [63]. The spatiotemporal distribution plots in the figures were generated using the python library Matplotlib [64]. The colors indicate the concentration gradient from zero (blue) to the maximum value of protein concentration (red) during the cell cycle. A disadvantage of such a plot is that a shallow gradient can be interpreted as significant changes in protein activity and localization. On the other hand, a very steep gradient can result in underestimation of fluctuations in protein activity and localization occurring at the lower range of concentration values. To avoid these problems and to make comparison between wild-type and mutant simulations more convenient, the color bar for each simulation indicates the concentration gradient from zero (blue) to maximum wild-type concentration *C* ^wt_max^ (red). A summary of all our simulation results is provided in S5 Table. The MATLAB code used to simulate the model is available at https://github.com/subkar/PleC_DivL_Spatial

## Results

### Contrasting ‘informal models’ of PleC function

While the dual roles of CckA as a phosphatase and kinase are acknowledged and understood [43–46,62], there are contrasting opinions regarding the function of the histidine kinase/phosphatase, PleC, in predivisional cells. *In vitro* experiments revealed that the kinase form of PleC is up-regulated by DivK~P [42]. Based on this important finding, an informal model was proposed [42], suggesting that the DivJ-dependent increase in the level of DivK~P that occurs during the swarmer-to-stalked transition induces PleC to become a kinase. As a kinase, PleC phosphorylates PleD, which in turn initiates a pathway for stalked-cell development [65]. Furthermore, the informal model suggests that, in the predivisional cell, PleC remains a kinase until cytokinesis separates PleC and DivJ into separate compartments [54–56].

The alternate view–that PleC is a phosphatase in predivisional cells [53]–is part of the protection-by-dephosphorylation model developed to explain reactivation of DivL and phosphorylation of CtrA in the predivisional stage [45]. As CtrA~P level falls in the stalked cell, its inhibition of DNA replication is lifted. Within the timeframe of the natural *Caulobacter* cell cycle, the single chromosome replicates only once to give two chromosomes. However, if cell division is blocked and the cell grows further, only the chromosomal origin that is proximal to the old pole begins a second round of replication to give a third chromosome [66]. This observation suggests a gradient of CtrA~P is established in the predivisional cell, with high concentration at the new pole (the incipient swarmer pole) and low concentration at the old pole (bearing the stalk). Mathematical modeling [46] has shown that such a phosphorylation gradient can only be established if CckA functions as a kinase at the new pole and a phosphatase at the old pole. This scenario requires DivL to be active at the new pole, i.e., unbound to DivK~P. Intriguingly, DivK~P is found to co-localize with DivL and PleC at the new pole. How does DivL remain unbound and active (up-regulating CckA kinase) while in close-proximity to DivK~P? The protection-by-dephosphorylation model suggests that predivisional PleC at the new pole is a phosphatase that is continuously working to dephosphorylate DivK~P and create an inhibition-free ‘protection zone’ for DivL [45].

**Fig 2:**
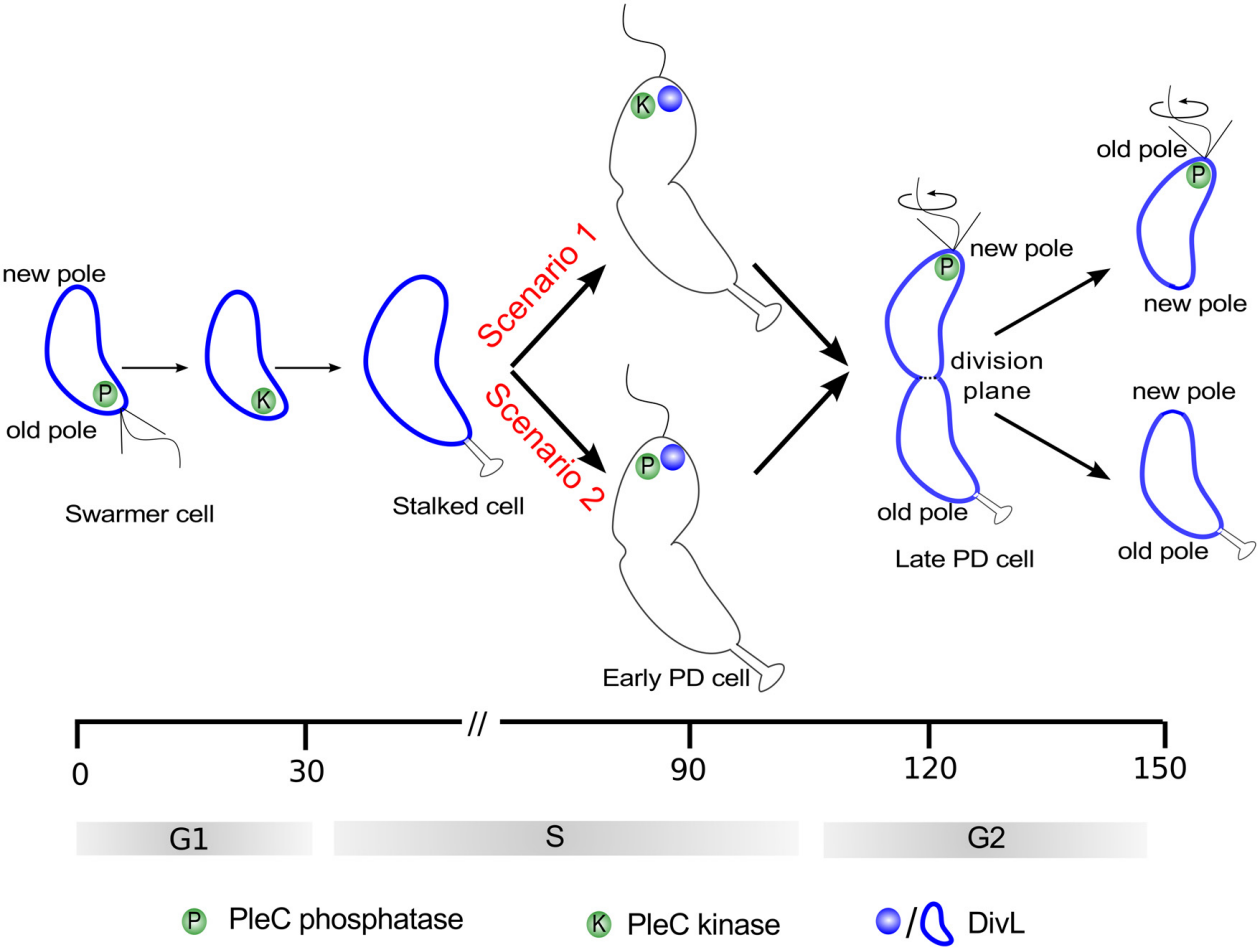
Two scenarios for the function of PleC (kinase or phosphatase) in the early predivisional cell. Spatiotemporal dynamics of PleC (green) and DivL (dark blue) during the cell cycle under two scenarios for the functional transition of PleC. In scenario 1, PleC is a kinase in early PD cells, as suggested in [54–56]. In scenario 2, PleC is a phosphatase in early PD cells, as described in the protection by dephosphorylation model [45,53].

Of these informal models, the first (in support of PleC kinase) is necessary to explain stalk formation, while the second (in favor of PleC phosphatase) posits conditions that are to be satisfied for replicative asymmetry. While the first model lays out changes of PleC function throughout the cell cycle, the second only addresses the function of PleC in the predivisional cell. Since PleC-dependent phosphorylation of PleD is required for development of the stalk, it is fair to assume that PleC is a kinase in the stalked cell. Hence, the difference in the two informal models can be narrowed down to the suggested function of PleC at the new pole of the predivisional cell (Fig 2). We use our spatiotemporal mathematical model to simulate changes of PleC function during the course of the cell cycle, in order to test the two conflicting theories.

### Rapid diffusion of DivK indicates that PleC is a kinase in the predivisional cell

In swarmer cells, PleC is localized at the old pole and functions as a phosphatase [20,58]. During the swarmer-to-stalked transition, PleC becomes a kinase [42,54,55] before it is cleared from the old pole in mature stalked cells [58]. Later in the cell cycle, PleC localizes at the new pole (the incipient swarmer pole). In our model, the transition from newborn swarmer cell to compartmentalized predivisional cell takes 150 minutes, a doubling time that is consistent with growth in poor medium [14,39,67,68].

Although there is considerable variation in the timing of various developmental transitions [53], the *Caulobacter* cell cycle appears to be robust to these variations. Therefore, in this deterministic model of cell cycle transitions, we assign time intervals for each stage of the cell cycle from generic descriptions of cell cycle progression [14]. Hence, in our model, the cell is in the swarmer stage for the first 30 min of the cell cycle and then in the stalked stage for the next 60 min (*t* = 30–90 min). PleC is localized at the old pole during the swarmer stage (*t* = 0–30 min). We assume the PleC remains at the old pole for the first 20 min (*t* = 30–50 min) of the stalked stage, because PleC and DivJ are known to be co-localized there for a short time in the developing stalked cell [54,55]. Although this 20-minute window may be on over-estimate, we chose it so that one can clearly see the transition of PleC to the kinase form before it is cleared from the stalked cell. (A shorter residency time does not qualitatively affect our simulation results; see S1 Fig). For *t* = 50–90 min, PleC is delocalized before it relocates to the new pole to define the start of the predivisional stage of the cell cycle (*t* = 90–150 min). We enforce compartmentalization at *t* = 120 min by preventing the diffusion of proteins across the mid-cell line. To distinguish between pre- and post-compartmentalized stages, we refer to these two stages as early predivisional (*t* = 90–120 min) and late predivisional (*t* = 120–150 min).

**Fig 3:**
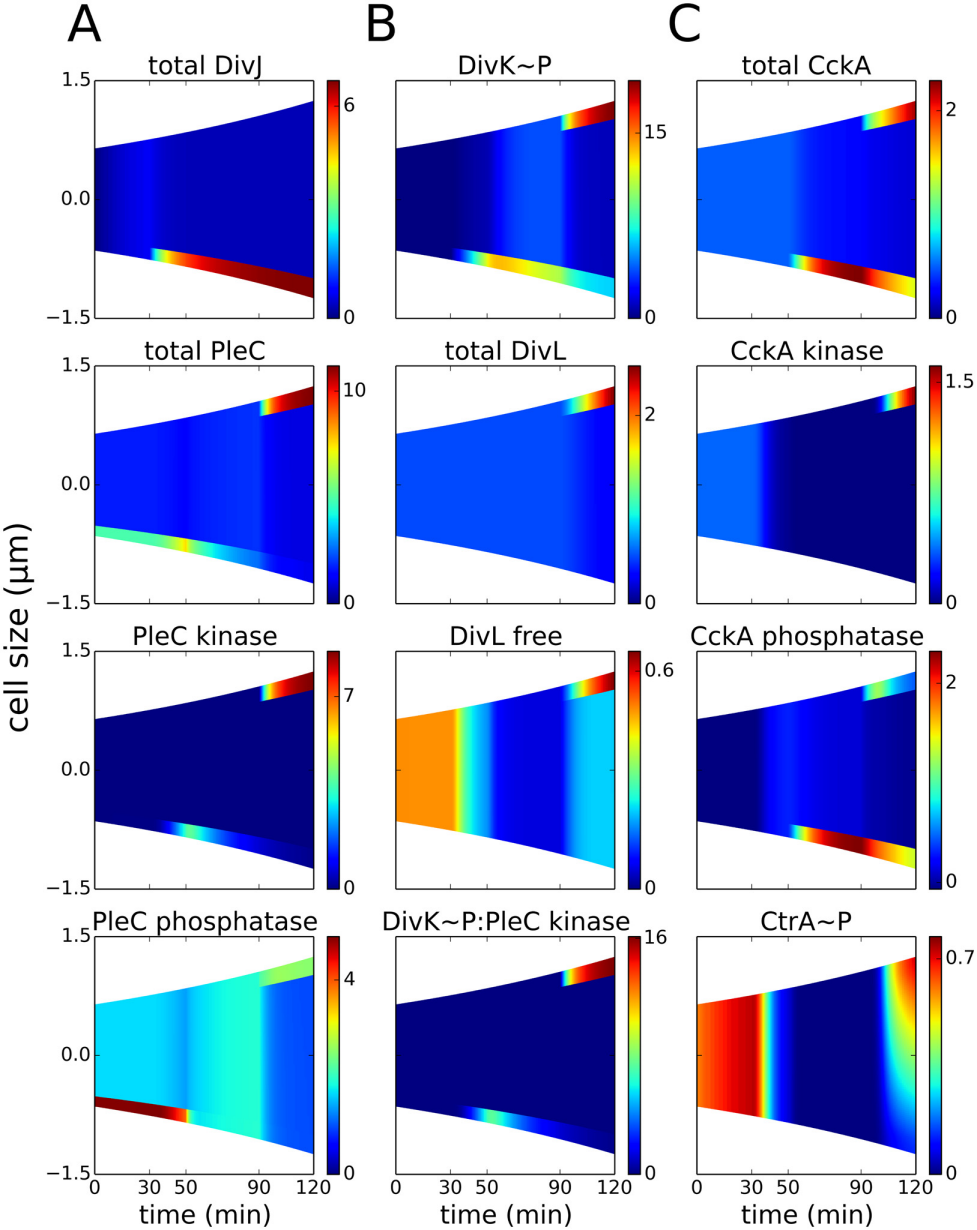
Co-localization of PleC kinase and DivL in the early predivisional cell is required for DivL reactivation. Spatiotemporal distributions of proteins during the cell cycle (prior to cytokinesis at *t* = 120 min). Color indicates concentration gradients from minimum (blue) to maximum (red). **(A)** DivJ is localized at the old pole (*t* = 30–120 min). The location of PleC is shifted from the old pole (*t* = 0–50 min) to the new pole of the predivisional cell (*t* = 90–150 min). Following DivJ localization, the function of PleC changes from a phosphatase to a kinase. **(B)** Upon phosphorylation, DivK localizes to the poles of the cell. Despite the presence of DivK~P at the new pole of the predivisional cell, DivL is present in the free form (unbound to DivK~P) because DivL co-localizes with PleC kinase and PleC kinase sequesters DivK~P, preventing it from binding to DivL. **(C)** CckA is uniformly distributed in the swarmer stage and localized at both poles in the predivisional stage. Reactivation of DivL at the new pole results in new-pole CckA becoming a kinase, while old-pole CckA remains a phosphatase. Consequently, the late predivisional cell establishes a gradient of CtrA~P along its length from high at the new pole to low at the old pole.

To initiate the swarmer-to-stalked transition, we localize DivJ to the old pole at *t* = 30 min (Fig 3A). The resultant surge in the level of DivK~P up-regulates the kinase form of PleC. In our simulations, when PleC translocates to the new pole, it continues to function as a kinase. Fluorescence-loss-in-photobleaching (FLIP) experiments show that DivK shuttles from pole to pole in about 5 seconds, indicating that the diffusion coefficient of DivK is 20–100 μm^2^ min^-1^ [53]. In our simulations, we assume that *D*_DivK_ = 100 μm^2^ min^-1^ for both DivK and DivK~P. In the absence of cytokinesis, DivK diffuses freely throughout the cell, from the new end, occupied by PleC, to the old end, containing DivJ. As a result, DivK~P, phosphorylated by DivJ at the old pole, is able to interact with new-pole PleC and induce it to become a kinase.

We investigated whether a smaller value of *D*_DivK_ would permit PleC to be a phosphatase in predivisional cells. For PleC to be a kinase in an incipient stalked cell and regain phosphatase activity in the predivisional cell, *D*_DivK_ had to be 1000-fold smaller than our estimated value (S2A Fig). Our simulations support the notion that, provided *D*_DivK_ is sufficiently small, the phosphatase form of PleC can create a “protection zone” for DivL by dephosphorylating DivK in the vicinity of the new pole (S2B Fig). Consequently, a gradient of CtrA~P can be established to enforce replicative asymmetry (S2C Fig). However, unlike the distribution pattern observed in experiments [20], we find that DivK~P no longer localizes at the new pole of predivisional cells. This aberrant result, combined with the fact that DivK~P would have to diffuse significantly more slowly than estimated from the experiments in [53], leads us to conclude that new-pole PleC cannot function primarily as a phosphatase. Hence, the protection-by-dephosphorylation model may not be the correct explanation for DivL reactivation in predivisional cells.

### PleC kinase can sequester DivK~P from DivL

If new-pole PleC is not acting as a phosphatase in early predivisional cells, how is DivL protected from DivK~P-dependent inhibition? Simulation results from our temporal model (S3 Fig) show that, in the process of up-regulation of the kinase form of PleC by its allosteric ligand DivK~P, a significant fraction of DivK~P is bound to PleC kinase. In contrast, DivK does not form a complex with PleC phosphatase that is prevalent in swarmer cells (S3 Fig). This simulation result is in agreement with fluorescence-resonance-energy-transfer (FRET) microscopy measurements that show interaction between DivK and PleC at the new pole [53]. These results would make sense if new-pole PleC is primarily a kinase, since DivK~P is an allosteric ligand that needs to remain bound to PleC to maintain it in the kinase form.

Based on these observations, we suggest that polar localization of DivK~P at the new pole of a predivisional cell may be a result of PleC being in the kinase form. In the predivisional cell, DivK~P is localized at both old and new poles [20]. Apart from PleC kinase, the only other recognized binding partners for DivK~P are DivJ and DivL [44]. DivJ accounts for old-pole localization of DivK~P, but DivJ’s absence from the new pole implies that it is not a binding factor for new-pole DivK~P. DivL is present at the new pole, but we require that DivL should not be bound to DivK~P, because DivL is actively up-regulating CckA kinase at the new pole. By ruling out DivJ and DivL and refraining from invoking an unidentified binding partner, we conclude that PleC kinase is the binding partner for new-pole DivK~P. Further, we hypothesize that PleC kinase outcompetes DivL for DivK~P binding. Instead of functioning as a phosphatase and dephosphorylating DivK~P, we predict that PleC is a kinase that sequesters DivK~P away from DivL. We speculate that DivK~P sequestration (rather than DivK~P dephosphorylation) may be the real reason for a “protection zone” for DivL at the new pole, so that DivL regains the ability to up-regulate CckA kinase there, prior to cytokinesis.

Using our model of the DivJ-PleC-DivK + DivL-CckA-CtrA network, we studied the plausibility of our inhibitor-sequestration hypothesis. As in the case of PleC, there is limited information on the mechanism behind the dynamic localization of DivL. Hence, we force DivL to spread throughout the cell initially (from *t* = 0 to *t* = 90 min), and later to localize at the new pole in the early predivisional stage (*t* = 90–120 min). Under these circumstances, our simulations show that: (1) DivL is active (unbound to DivK~P) in the swarmer stage (Fig 3B); (2) during the swarmer-to-stalked transition, the level of active DivL falls, as it binds with DivK~P; and (3) in the early predivisional stage (*t* = 90–120 min), DivL is localized at the new pole in the active form, even though DivK~P is present at the same location. These simulations confirm our hypothesis that, at the new pole of an early predivisional cell, where PleC, DivK and DivL co-localize, PleC kinase sequesters DivK~P, allowing DivL to be reactivated and CckA to be a functional kinase.

Why does inhibitor sequestration fail to protect DivL in the stalked cell (*t* = 50–70 min), when PleC is transiently localized at the old pole as a kinase? We reason that the spatial separation of PleC kinase and DivL in the stalked cell stage means that most DivL molecules lie outside the protection zone created by PleC-dependent inhibitor sequestration. Moreover, by spreading over the entire length of the cell, DivL can be inhibited by a lower concentration of DivK~P per unit length of the cell. Our simulations demonstrate that *Caulobacter* cells can program different cell fates by regulating DivL inhibition through spatial reorganization of DivL and PleC.

### The inhibitor-sequestration model reproduces replicative asymmetry in the early predivisional cell and swarmer pole development in the late predivisional cell

**Fig 4:**
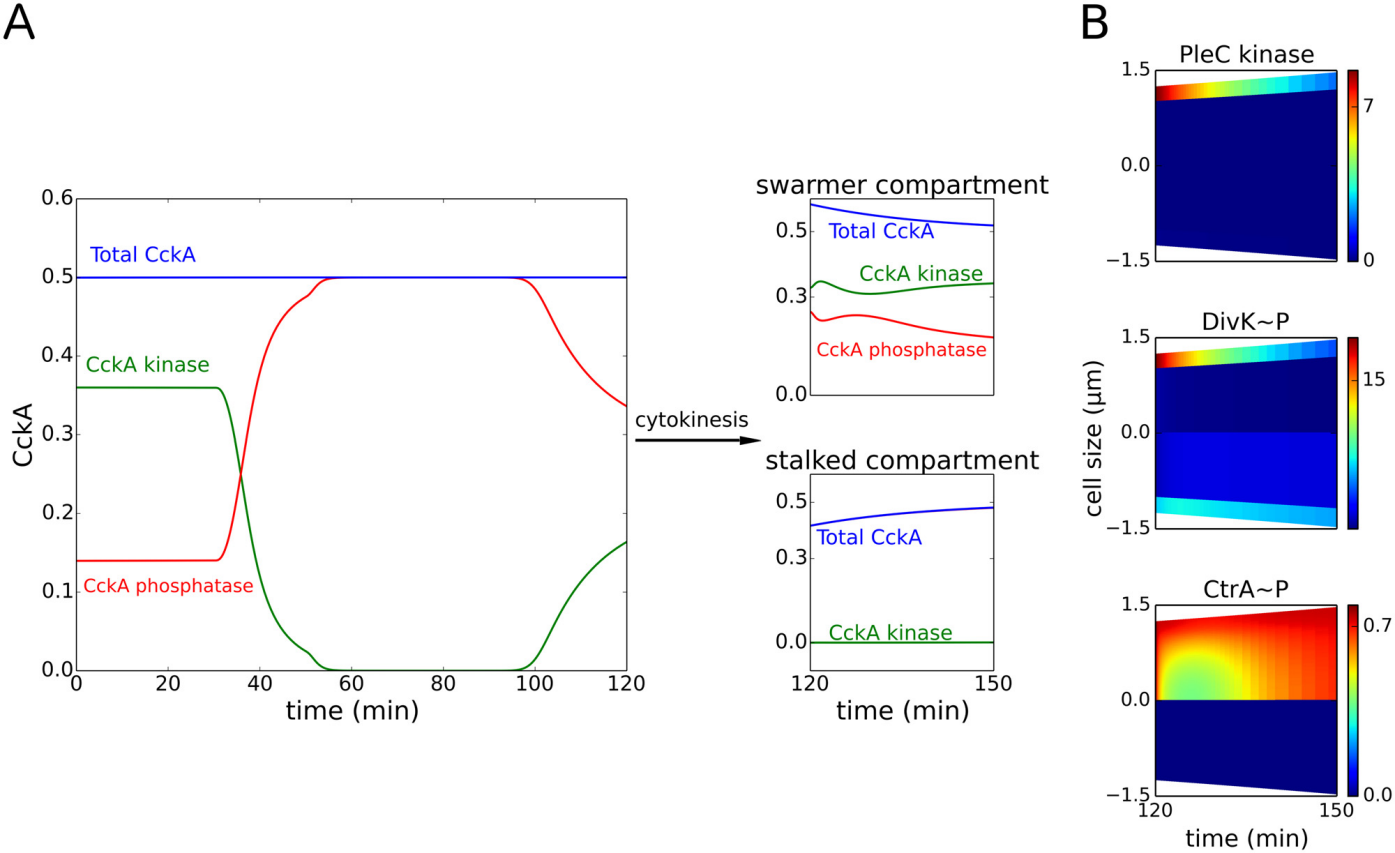
Following cytokinesis, PleC reverts to the phosphatase form. **(A)** The total concentration of CckA (blue curve) remains constant during the cell cycle. However, the proportions of phosphatase (red curve) and kinase (green curve) forms of CckA change for each stage of the cell cycle. After cytokinesis (compartmentalization), the concentrations of the kinase and phosphatase fractions of CckA in the swarmer and stalked compartments (*t* = 120–150 min) are similar to their concentration in the non-compartmentalized swarmer (*t* = 0–30 min) and stalked cell stages (*t* = 30–90 min), respectively. **(B)** Spatiotemporal distribution of PleC kinase, DivK~P and CtrA~P after compartmentalization (at *t* = 120 min). Color indicates concentration gradients from minimum (blue) to maximum (red).

CckA localization is governed by both PopZ [69] and DivL [61]. Although the spatial distribution of CckA shows cell-to-cell variability, the consensus opinion is that CckA protein is spread uniformly throughout the cell in the swarmer stage, followed by old-pole localization in the stalked cell, followed by a bipolar distribution in the early predivisional stage [52,70]. According to our model simulations, the spatiotemporal distribution of CckA activity reflects changes in local concentrations of free DivL. In the swarmer cell, when CckA is uniformly distributed, 70% of CckA is in the kinase conformation (Figs 3C and 4A). In the stalked cell (*t* = 30–90 min), DivL inactivation results in an increase in the phosphatase fraction of CckA, even as total CckA localizes to the old pole. Finally in the early predivisional cell (*t* = 90 min), a second focus of CckA co-localizes as a kinase with reactivated DivL, while old-pole CckA remains in the phosphatase form (Fig 3C). At the early predivisional stage, less than half (~36%) of total cellular CckA is in the kinase conformation (Fig 4A). Importantly, however, this 36% is localized in the incipient swarmer half (new pole) of the early predivisional cell (Fig 3C).

It has been suggested that replicative asymmetry is a result of differential phosphorylation and dephosphorylation of CtrA across the length of the predivisional cell [46]. Synthesis and degradation of CtrA has no bearing on the CtrA phosphorylation gradient. For these reasons, we do not account in our model for changes in the total amount of CtrA protein by means of transcriptional [37,40,71] and proteolytic [72–74] controls. We assume that CtrA is synthesized in the unphosphorylated form at a constant rate, while CtrA and CtrA~P are degraded at a rate proportional to their individual concentrations. Fluorescence-recovery-after-photobleaching (FRAP) experiments indicate that CtrA has a diffusion coefficient of 60–600 μm^2^ min^-1^, while modeling studies suggest that, in order to obtain a gradient of CtrA~P, the rate constants for phosphorylation and dephosphorylation must be faster than the inverse diffusive time scale (*k*_ctr_phos_ = *k*_ctr_kin_ >> 2*D*/*L* ^2^) [46]. In our model, we assume *D*_CtrA_ = *D*_CtrAP_ = 100 μm^2^ min^-1^ and *k*_ctr_phos_ = *k*_ctr_kin_ = 600 min^-1^. Our simulations show that CtrA~P is dephosphorylated during the swarmer-to-stalked transition. Once CckA localizes as a kinase at the new pole, a gradient of CtrA~P is established across the cell body (Fig 3C).

In order to simulate compartmentalized predivisional cells (*t* = 120–150 min), we use end-point concentrations from the early predivisional stage (*t* = 120 min) as initial conditions in a simulation of a growing cell with a diffusion barrier at mid-cell. Since DivJ and PleC are in separate compartments, the level of new pole PleC kinase in the swarmer compartment begins to decline and DivK becomes unphosphorylated (Fig 4B). The concentrations of the kinase and phosphatase forms of CckA in the swarmer and stalked compartments at *t* = 150 min are almost identical to their concentrations during the swarmer (*t* = 0–30 min) and stalked stages (*t* = 30–90 min), respectively (Fig 4A). Finally, CtrA in the swarmer compartment of the late predivisional cell is phosphorylated, while stalked-compartment CtrA is unphosphorylated. While replicative asymmetry does not require cytokinesis, our simulations show that compartmentalization reinforces cell fate asymmetry by deactivating PleC kinase and dephosphorylating DivK~P in the swarmer compartment.

### DivK overexpression negates inhibitor sequestration

**Fig 5:**
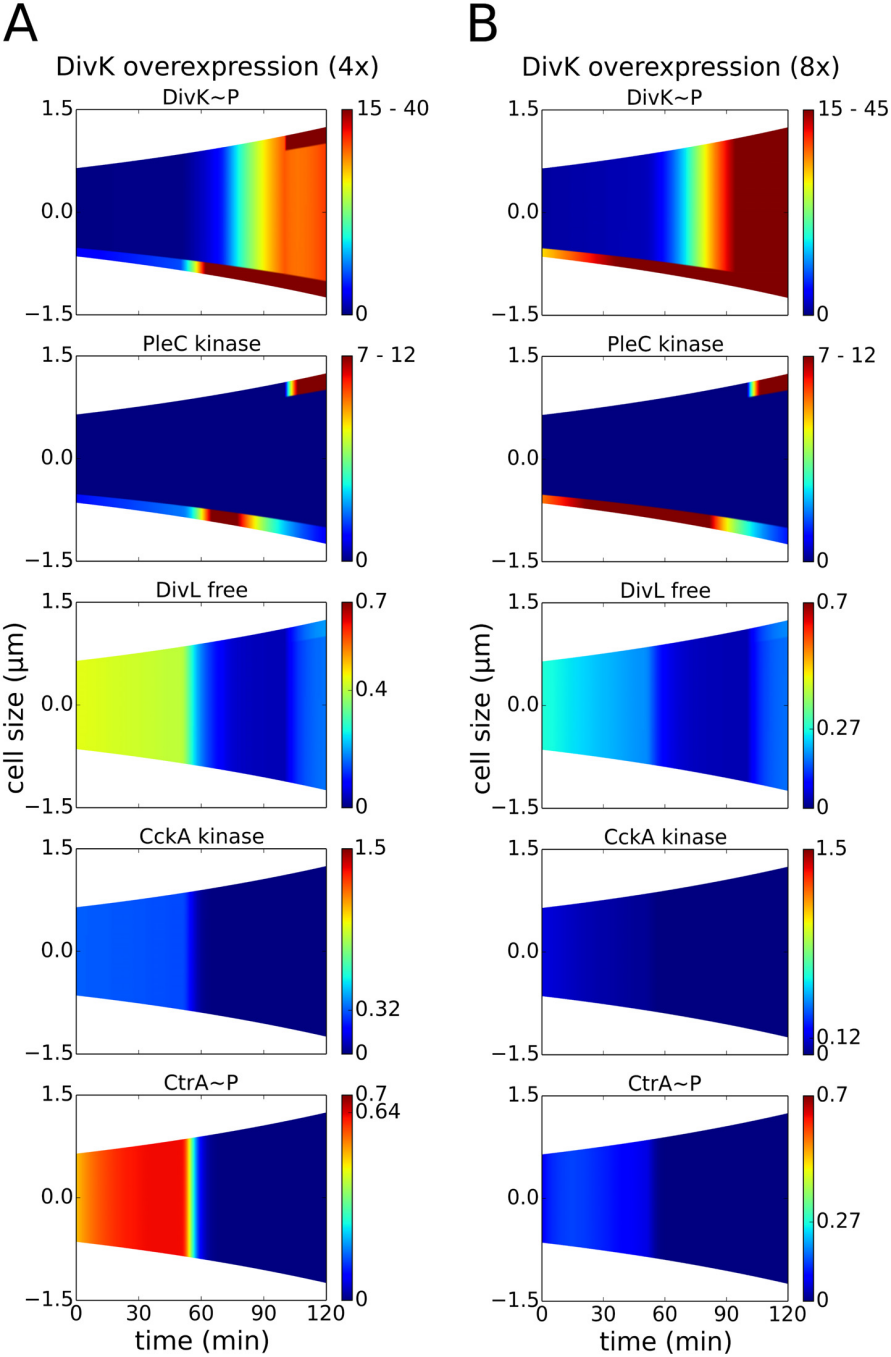
Overexpression of DivK may prevent the cell from progressing though different developmental stages. For better comparison of mutant and wild-type distributions, the colors indicate concentration gradients from zero (blue) to maximum wild-type concentration, *C* ^wt_max^ (red). For *C* ^wt_max^ < *C* ^mut_max^, the red color represents all concentrations from *C* ^wt_max^ to *C* ^mut_max^. For *C* ^wt_max^ > *C* ^mut_max^, an additional tick between 0 and *C* ^wt_max^ indicates maximum concentration in the mutant. **(A)** A four-fold increase in DivK synthesis (*k*_syn_ = 0.2 min^-1^) prevents competition between DivL and PleC kinase. DivL is not reactivated in the predivisional stage, leading to the loss of CckA kinase and CtrA~P asymmetry in the early predivisional stage. **(B)** An eight-fold increase in DivK synthesis (*k*_syn_ = 0.4 min^-1^) induces PleC to become a kinase independent of DivJ. Hence, the swarmer stage of the cell cycle is circumvented.

The main assumption of our model is that PleC kinase and DivL compete for binding DivK~P. In simulations of a wild-type cell, PleC kinase at the new pole of a predivisional cell outcompetes DivL for binding DivK~P, thus allowing DivL to remain in its active conformation. Overexpression of DivK should undermine this mechanism, since there will be sufficient DivK~P to bind both PleC kinase and DivL. Upon increasing DivK synthesis by four-fold (*k*_syn_ = 0.2 min^-1^), we see an increase in the level of PleC kinase in both the stalked and predivisional cell compared to the wild-type simulation (Fig 5A). Excess DivK~P in the over-expressing cell binds to and inhibits DivL. Consequently, in the predivisional cell, new-pole CckA does not convert to its kinase form, and CtrA does not get re-phosphorylated.

In our temporal model [57], we showed that the phosphatase-to-kinase transition of PleC is thermodynamically more favorable when DivK~P, not unphosphorylated DivK, is the allosteric ligand. However, *in vitro* experiments demonstrated that DivK need not be phosphorylated to up-regulate PleC kinase [42]. We reasoned that the concentration of DivK *in vivo* is within a range that requires DivK to be phosphorylated in order to induce the PleC kinase conformation. In this case, the swarmer-to-stalked transition is controlled by a bistable PleC switch that is flipped to the kinase state by the action of DivJ. When the rate of synthesis of DivK was increased 5–10 fold, our temporal model predicted that PleC kinase would be up-regulated even in the absence of DivJ-dependent phosphorylation of DivK. In this scenario, PleD would be phosphorylated throughout the cell cycle, thus committing the cell to obligate stalked cell morphology.

In our current spatiotemporal model, a four-fold increase in the rate of DivK synthesis results in the cell being unable to enter the predivisional stage of the life cycle. However, because PleC is a phosphatase early (*t* = 0–50 min) and a kinase later (*t* = 50–120 min) in the simulation, the cell is predicted to retain distinct swarmer and stalked stages (Fig 5A). Increasing the rate of synthesis of DivK by eight-fold (*k*_syn_ = 0.4 min^-1^), we find (Fig 5B) that PleC is a kinase even in the absence of DivJ (*t* = 0–30 min). DivK is phosphorylated and localized to the old pole, while the level of free and active DivL falls to a third of its wild-type concentration. Throughout the cell cycle, CckA is a phosphatase and CtrA is unphosphorylated.

These simulation result are consistent with the experimentally observed phenotype of a *divK* overexpressing strain, namely, chromosome accumulation, and significant reduction in the level of CtrA~P and autophosphorylated CckA [44,50]. In addition, our model suggests that the cell shows a graded response to *divK* overexpression. A four-fold increase in DivK sees the cell retain swarmer-to-stalked transition, but lose predivisional cell asymmetry on account of being unable to rephosphorylate CtrA. Further increase (eight-fold) restricts the cell to a stalked-only morphology, since PleC becomes a kinase and DivK is phosphorylated independent of DivJ.

### Co-localization of PleC and DivL is essential to DivL activity

**Fig 6:**
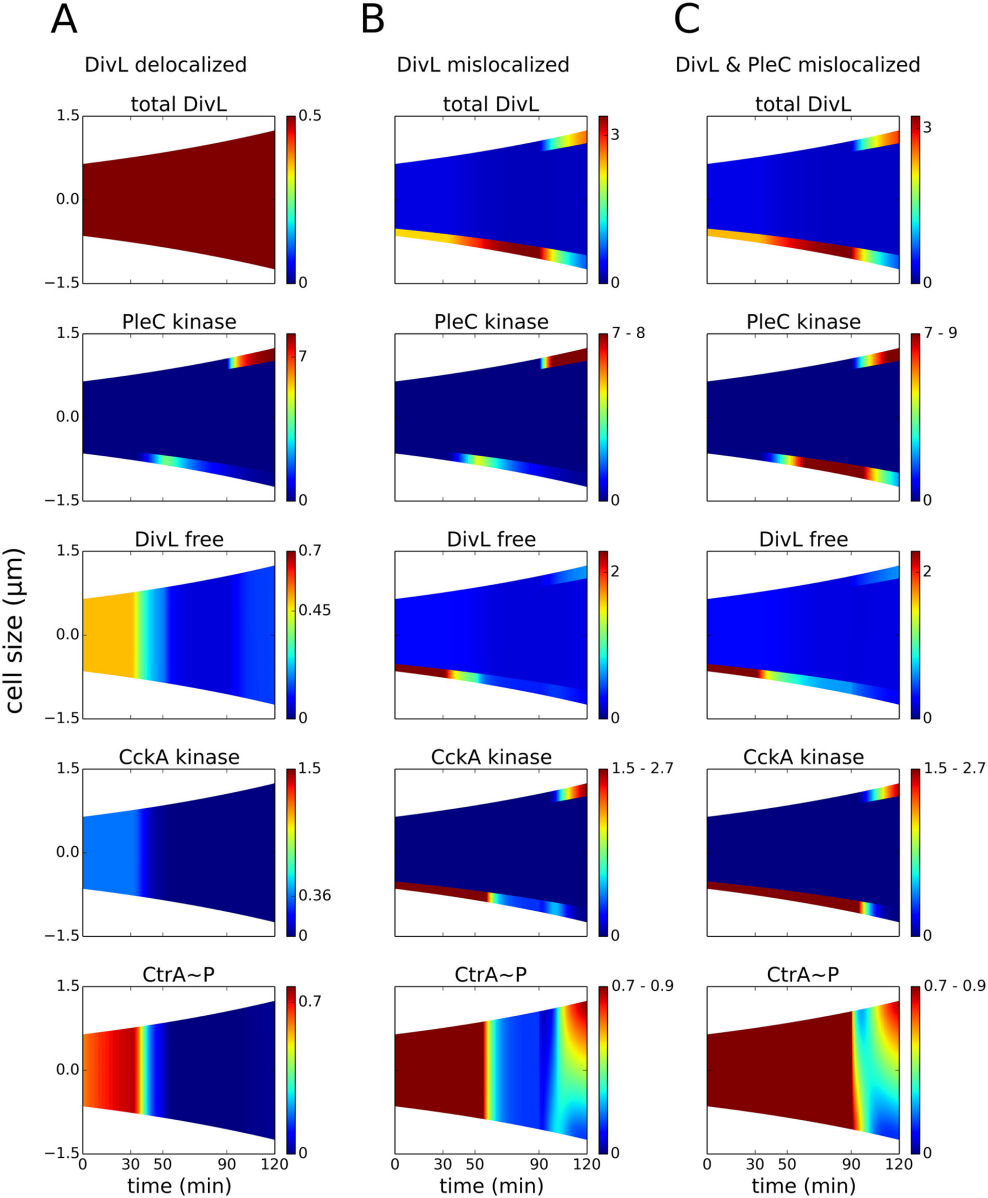
Swarmer-to-stalked transition is uncoupled from G1-to-S transition in DivL mislocalization mutants. Concentration gradients are color coded as in Fig 5. **(A)** DivL is uniformly distributed at all times in the cell cycle. Even though PleC transitions to a kinase, DivL remains deactivated (bound to DivK~P) in the predivisional cell, resulting in no gradient of CtrA~P. **(B)** DivL (and CckA) are localized at the old pole (*t* = 0–90 min), before switching to the new pole in the predivisional cell (*t* = 90–120 min). Even after PleC transitions to a kinase, DivL is not deactivated in stalked cells, delaying the dephosphorylation of CtrA~P until PleC delocalizes from the old pole (at *t* = 50 min). **(C)** DivL and PleC are co-localized at one of the poles during all stages of the cell cycle. Hence, CtrA is phosphorylated through all stages of the cell cycle (G1-arrest).

*divL* mutant cells that cannot localize DivL at the new pole in the predivisional stage also fail to localize and activate CckA [61]. In contrast, in the swarmer stage, uniformly distributed DivL is actively up-regulating CckA. This lack of a causal relationship between localization and activation across two stages of the cell cycle indicates that new-pole localization alone cannot account for DivL activity. Rather, it appears that the act of localizing to the new pole protects DivL from inhibition by DivK~P in the predivisional cell. This may seem counter-intuitive at first, since DivL is positioned in close proximity to its inhibitor DivK~P in the early predivisional cell. We contend that the spatial segregation of PleC and DivL in stalked cells enables DivK~P to bind and inhibit DivL. In contrast, DivL is protected in predivisional cells by co-localized PleC kinase, which sequesters the DivL-inhibitor, DivK~P. To test this hypothesis, we simulate a scenario where DivL and CckA are uniformly distributed (delocalized) throughout the cell cycle. Consistent with our reasoning, the simulations show (Fig 6A) that free and active DivL is present only in the swarmer stage when no DivK~P is present. Despite the presence of PleC kinase at the new pole during the predivisional stage, DivK~P is able to inhibit DivL as it is diffusely spread over the entire cell. Consequently, new-pole CckA is a phosphatase, and a CtrA~P gradient is not established. Based on this simulation result, we propose that new-pole localization of DivL is not solely responsible for its activation. Instead, it is the co-localization of DivL and PleC kinase that enables DivL to up-regulate CckA kinase. The situation of delocalized DivL and CckA has been encountered in experiments where DNA replication is blocked, and, as in our simulations, these experiments reveal that CckA retains phosphatase activity and CtrA remains unphosphorylated [62].

At the other extreme, we simulated a mutant in which DivL is always localized at one of the poles (Fig 6B). In this mislocalization mutant, DivL is initially present at the old pole of swarmer and stalked cells (*t* = 0–90 min), and later relocates to the new pole in predivisional cells (*t* = 90–120 min). In comparison to the wild-type case, the level of free DivL is higher for a further 20 min, from *t* = 0 to *t* = 50 min, which is also the duration of PleC localization at the old pole. While the PleC phosphatase-to-kinase transition occurs as usual (*t* = 30 min), DivL inactivation and the consequent dephosphorylation of CtrA is delayed by a period consistent with the co-localization of PleC kinase and DivL. Once PleC is delocalized (*t* = 50 min), DivL activity drops, CckA reverts back to a phosphatase, and CtrA is dephosphorylated. Essentially, the novel mutant is characterized by a delay in G1-to-S transition, which otherwise occurs concurrent to the swarmer-to-stalked transition. To further emphasize this point, we simulated the case where PleC and DivL are retained at the old pole throughout the stalked stage, before being redistributed to the new pole in the predivisional cell (Fig 6C). PleC becomes a kinase in the stalked and predivisional cell. However, since DivL and PleC are always co-localized in this novel mutant, CckA remains a kinase and CtrA is phosphorylated during all stages of the cell cycle.

Overall, our simulations suggest that *Caulobacter* cells exploit DivL and PleC localization to fashion two separate phosphorylation profiles for CtrA in stalked and predivisional stages of the cell cycle. Furthermore, a uniform distribution of DivL is essential for the temporal coupling of the swarmer-to-stalked transition with the G1-to-S transition.

### Loss of asymmetry is a physiological consequence of mutations that alter the spatiotemporal profile of inhibitor sequestration

If the kinase form of PleC is required for inhibitor sequestration, then it follows that *pleC* mutants without kinase function would be ill-equipped to resolve the stalked and predivisional stages of the cell cycle. This class of mutants includes Δ*pleC*, *pleC*_H610A_, *pleC*_F778L_ and *divK*_D90G_. The first two mutations (Δ*pleC* and *pleC*_H610A_) produce non-motile, pili-less and stalk-less cells [20]. *pleC*_F778L_ mutants are similar to wild-type cells, except that they produce underdeveloped stalks [42]. On the other hand, *divK*_D90G_ mutant cells are arrested in G1 and lose morphological asymmetry [53]. We sought to explain the physiology of these mutants from the perspective of our inhibitor-sequestration hypothesis.

**Fig 7:**
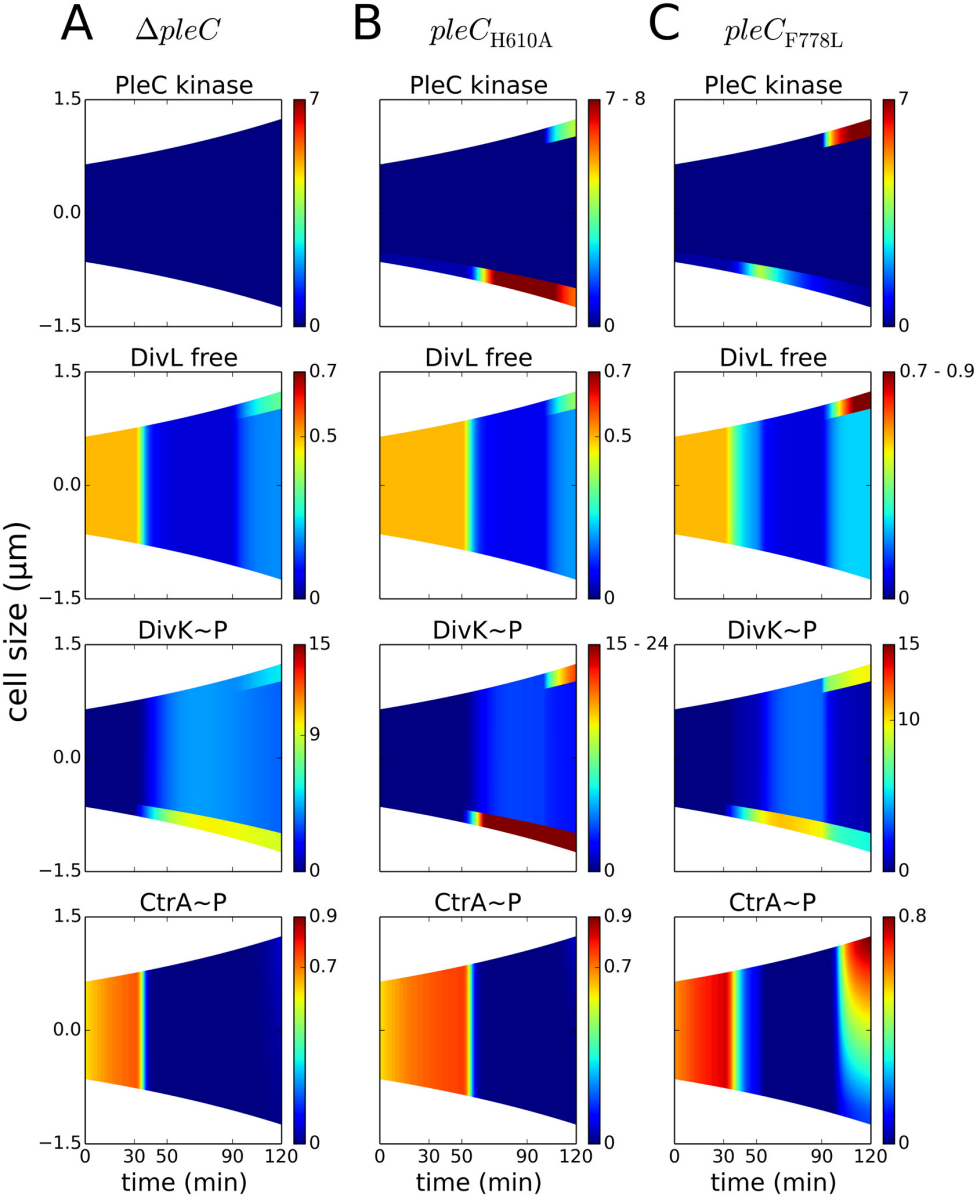
PleC kinase conformation is required to establish replicative asymmetry. Concentration gradients are color coded as in Fig 5. **(A)** In Δ*pleC* mutant cells (K^-^P^-^B^-^), free active form of DivL is lower than in wild-type cells, resulting in loss of CtrA~P in the predivisional cell. **(B)** In *pleC*_H610A_ mutant cells (K^-^P^-^B^+^), an elevated level of DivK~P results in less active form of DivL and reduced CtrA~P. **(C)** In *pleC*_F778L_ mutant cells (K^-^P^+^B^+^), inhibitor sequestration is retained, resulting in a normal CtrA~P gradient.

To simulate Δ*pleC* mutant cells, we set the rate constant for PleC synthesis to zero (S3 Table). In this case, the cell completes the G1-to-S transition but fails to progress any further (Fig 7A). Devoid of PleC kinase, PleD will not be phosphorylated, resulting in the stalk-less phenotype. In the predivisional cell, the kinase form of PleC is not available to sequester DivK~P. Early in the cell cycle, DivK~P localizes to the old pole by binding to DivJ, and later DivK shows moderate bipolar localization through binding to DivL at the new pole. Consequently, DivL remains inactive at the new pole of predivisional cells, and the cell does not exhibit replicative asymmetry.

*pleC*_H610A_ encodes a mutant protein that is both phosphatase- and kinase-negative (K^-^P^-^). For reasons unknown, PleC_H610A_ is not released from the old pole of stalked and predivisional cells [20]. Hence, we enforce bipolar localization of PleC in our simulations of this mutant (S4 Table). While the enzyme is incapable of auto-phosphorylation or phosphotransfer, it is unknown if PleC_H610A_ retains its ability to undergo allosteric modification. We parameterize the *pleC*_H610A_ mutant such that PleC_H610A_ undergoes allosteric modifications by DivK but is unable to auto-phosphorylate or change the phosphorylation status of DivK (S3 Table). Under these assumptions, our simulations show that PleC transitions from an inactive phosphatase form to an inactive kinase form during the swarmer-to-stalked transition (Fig 7B). Although the inactive kinase form of PleC sequesters DivK~P (according to our assumptions), DivL is nonetheless inactivated. The reason why inhibitor sequestration fails in this case is the inability of PleC_H610A_ to dephosphorylate DivK. As a result, the level of DivK~P is high in these mutant cells, and the excess DivK~P molecules bind to and inactive DivL. The failure to dephosphorylate new pole DivK~P, which is characteristic of Δ*pleC* and *pleC*_H610A_ mutants, is also reproduced in our simulations.

The two mutant alleles *pleC*_H610A_ and *pleC*_F778L_ are similar in all aspects except that PleC_F778L_ retains its phosphatase activity. Hence, in our simulations, new-pole PleC_F778L_, like PleC_H610A_, can acquire the kinase conformation and sequester DivK~P. Unlike PleC_H610A_ however, the ability of PleC_F778L_ to dephosphorylate DivK means that the level of DivK~P is low enough to allow competition between DivL and PleC kinase. In this scenario, DivL remains active and up-regulates CckA kinase. Hence, even though PleC_F778L_ kinase is unable to phosphorylate DivK, our simulations show that the inactive kinase form of PleC is present at the new pole, where it fulfills the important role of sequestering DivK~P (Fig 7C).

Based on our results, we propose that the kinase form of PleC has two independent and important functions to ensure normal progression through the *Caulobacter* cell cycle. Firstly, the kinase activity of PleC is required to phosphorylate PleD and initiate stalk development. Secondly, the kinase conformation enables PleC to bind and sequester DivK~P, an effect that is essential for DivL activity and the replication-asymmetry of the predivisional cell.

Under conditions of replication inhibition, CckA and DivL fail to localize at the new pole [62]. Because *divK*_D90G_ mutant cells arrest in G1 [53], we assume that they fail to localize CckA and DivL to the new pole. Hence, in our simulations of this mutant strain (S4 Fig), we enforce DivL and CckA to be delocalized in the predivisional cell. The molecular defects of the *divK*_D90G_ allele are the inability of DivK_D90G_ to up-regulate PleC kinase [42] and its reduced efficiency in binding and inhibiting DivL [45]. Consequently, in our simulations, PleC kinase level in the stalked and predivisional cell stages falls to a tenth of its wild-type maximum. The experimentally observed unipolar localization of DivK in *divK*_D90G_ mutant cells [53] is also reproduced in our simulations. Our results suggest that the lack of a new pole focus of DivK_D90G_ is due in part to its ineffective binding to DivL and also to the state of its second binding partner, PleC, which is in the phosphatase form and thus not strongly bound to unphosphorylated DivK_D90G_. The unipolar localization of DivK in *divK*_D90G_ cells further supports the notion that new-pole PleC in wild-type cells is in the kinase form. Even though PleC kinase is unavailable to sequester DivK_D90G_~P, DivL retains much of its activity, since the DivK_D90G_ mutant protein is an ineffective inhibitor of DivL. The level of active DivL remains high until *t* = 50 min, and only shows moderate decrease during the period when PleC is delocalized (*t* = 50–90 min). As a consequence, CtrA remains phosphorylated throughout the cell cycle, which explains the G1-arrest observed in experiments [72].

## Discussion

### A spatiotemporal model of cell cycle progression in *Caulobacter*

In earlier work, we proposed a mechanism for the DivK-dependent allosteric regulation of PleC kinase [57]. Our mathematical model of the proposed mechanism, based on elementary chemical reactions, showed that the transition of PleC activity from phosphatase to kinase might function as a bistable switch flipped by DivJ. We believe bistability of the PleC switch ensures a robust and irreversible transition from swarmer to stalked cell morphology. Based on our simulations of mutant phenotypes such as *divK*_D90G_ and *pleC*_F778L_, we predicted that the PleC kinase form is essential for stalked cell development. While the model itself was focused on understanding temporal dynamics during the window of the swarmer-to-stalked transition, we speculated that PleC at the new pole of predivisional cells is a kinase. Without an accurate spatiotemporal model however, we could not predict the effects that diffusion and differential localization might have on the behavior of the molecular switch or its physiological impact on the development of different stages of the asymmetric division cycle.

In this paper we present a spatiotemporal model of the network of coupled signaling pathways, DivJ-PleC-DivK and DivL-CckA-CtrA, which determine the phosphorylation status of CtrA in predivisional cells and hence the replicative asymmetry of the incipient swarmer and stalked cells. Our model extends earlier efforts to model various aspects of cell cycle control in *Caulobacter*. Spatiotemporal models focused solely on the DivJ-PleC-DivK [75] pathway or the CckA-CtrA [46] pathway have been developed. However, these models simulated only the predivisional stage of a non-growing *Caulobacter* cell. Hence, the proposed mechanisms investigated by these models could not be validated against the behavior of wild-type and mutant cells at other stages of the cell cycle. Other models have captured various temporal aspects cell cycle regulators [57,76,77], without considering spatial localization of the proteins. The reaction-diffusion model described here captures the spatiotemporal dynamics of the DivJ-PleC-DivK + DivL-CckA-CtrA network in a *Caulobacter* cell that grows from a newborn swarmer cell to the late predivisional stage. We use the model is to investigate our “inhibitor sequestration” hypothesis for generating a CtrA~P gradient in the predivisional cell, and to validate our hypothesis against the phenotypes of wild-type and mutant *Caulobacter* cells at every stage of the cell division cycle (S5 Table).

As with any dynamical modeling approach, trade-offs must be made in terms of the molecular details to be included in / neglected from the model, so that the phenomenon under study is accurately described without the model becoming unwieldy to optimize or constrain with experimental data. All the proteins under study in our model are subject to transcriptional regulation and are part of complex localization mechanisms; aspects that were not included in this study. For instance, the bifunctional histidine kinases (PleC and CckA) and their partners (DivJ and DivL) change their locations in the *Caulobacter* cell during succeeding stages of the division cycle by mechanisms that are poorly understood at present. Since an accurate mechanistic model of the localization of these proteins is not essential to answering the key questions proposed in this paper, we have enforced a set of localization-rules for these proteins and used our model to predict the consequences of these experimentally observed localization patterns on the phosphorylation states of the response regulators, DivK and CtrA.

Other important aspects that have been excluded from the present model are the transcriptional regulation of CtrA [40] and the ClpXP proteolytic machinery that degrades CtrA during the G1-to-S transition [78–80]. Since we were only concerned with changes in the phosphorylation status of CtrA as a measure of DivL and CckA activity, we chose to ignore the regulated synthesis and degradation of CtrA in our model. In any case, the cell cycle proceeds normally in mutant strains containing non-degradable but phosphorylable forms of CtrA [81], indicating that ClpXP-dependent degradation is non-essential as long as CtrA activity is modulated via phosphorylation. The level of total CtrA remains roughly constant during the replication-division cycle of these normally behaving mutant strains [81], an observation that we approximate by using basal rates for CtrA synthesis and degradation. Hence, we believe that our simulation results and the qualitative conclusions we draw would remain the same were we to include regulated CtrA synthesis and degradation.

### Inhibitor sequestration is a third function of the bifunctional histidine kinase PleC

We propose that PleC performs three distinct functions that are crucial to proper progression through the *Caulobacter* cell cycle. In the swarmer cell, PleC is a phosphatase that maintains DivK in its dephosphorylated state. In stalked cells, PleC is a kinase that phosphorylates PleD. Interestingly, the kinase conformation enables PleC to perform a third function—that of binding to DivK~P, which permits reactivation of DivL, activation of CckA at the new pole, and phosphorylation of CtrA in the swarmer-half of a predivisional cell.

**Fig 8:**
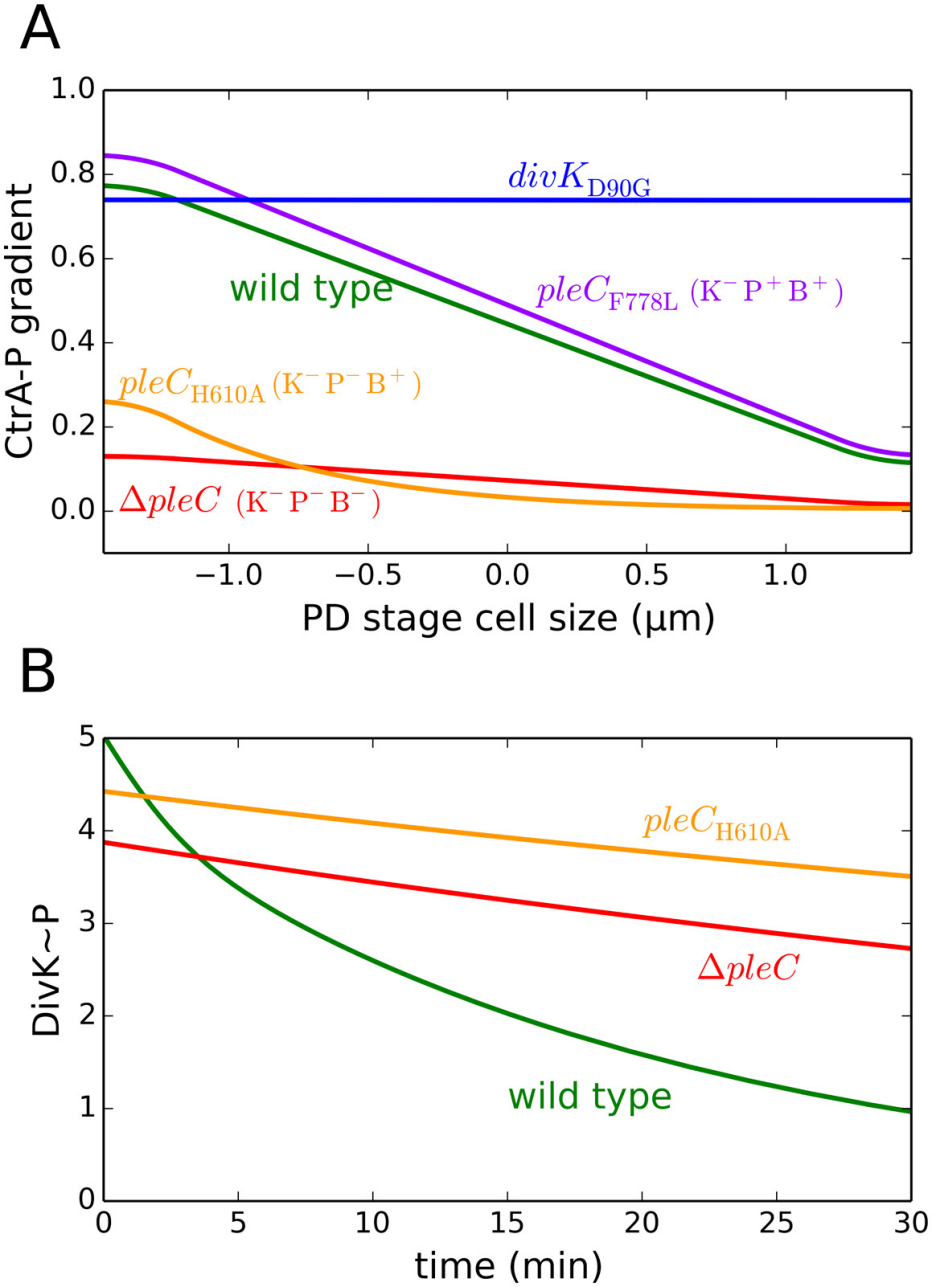
The model simulates distribution patterns of CtrA~P and DivK~P in mutant cells. **(A)** Model simulations predict the concentration gradient of CtrA~P prior to cytokinesis (*t* = 120 min) in mutant cells: *pleC*_F778L_ (purple), *divK*_D90G_ (blue), Δ*pleC* (red) and *pleC*_H610A_ (yellow). The distribution for the wild-type cells is plotted in green for reference. **(B)** The model reproduces the inadequate dephosphorylation of DivK~P observed in the swarmer compartment of post-compartmentalized cells in the Δ*pleC* (red) and *pleC*_H610A_ (yellow) mutant strains.

Our model reproduces the expected distribution pattern of CtrA~P in predivisional cells of *pleC* and *divK* mutants (Fig 8A). Each of these mutants can be defined in terms of the loss of one or more of the three distinct functions of PleC—namely, the auto-phosphorylation and phospho-transfer activities of the kinase form (K), the catalytic activity of the phosphatase form (P), and the DivK~P binding capability of the kinase form (B). A CtrA~P gradient from the new end (high) to the old end (low) is observed in wild-type cells (K^+^P^+^B^+^) that have all three functions. Mutants that are defective only in the kinase activity (K^-^P^+^B^+^) can establish a CtrA~P gradient, as in the case of *pleC*_F778L_. In this mutant, inhibitor sequestration is still operative, enabling DivL reactivation at the new pole of predivisional cells. In *pleC*_H610A_ (K^-^P^-^B^+^) mutant cells, the phosphatase activity is impaired as well, resulting in an increased level of DivK~P, which binds both PleC and DivL. Therefore, a CtrA~P gradient cannot be established, despite the retention of DivK~P’s binding function. In Δ*pleC* mutant cells, inhibitor sequestration is absent and DivK~P is also elevated, resulting in dephosphorylation of CtrA in the stalked and predivisional stages. We conclude that although auto-phosphorylation and phospho-transfer are dispensable, the phosphatase function of PleC and the DivK~P sequestration role of PleC kinase are required for replicative asymmetry.

### Delocalized DivL couples the morphological transition with the G1-to-S transition

The morphological transition that occurs during stalked-cell development requires PleC to be active as a kinase, whereas the G1-to-S transition requires deactivation of DivL. The two processes are coupled because PleC kinase phosphorylates DivK, and DivK~P binds to and inactivates DivL. Our simulations show that mislocalizing PleC has no bearing on its functional change from phosphatase to kinase. On the other hand, co-localizing DivL with PleC kinase in the stalked cell does not allow DivK~P to inhibit DivL; hence, CtrA~P level stays high and DNA replication is inhibited. This simulation result prompts us to suggest that the G1-to-S transition requires DivL to be uniformly distributed in the cell membrane. We predict that the G1-to-S transition would be uncoupled from the morphological swarmer-to-stalked transition in a novel mutant where DivL always co-localizes with PleC.

### Reversion of PleC to phosphatase form is required for swarmer cell development

At cytokinesis, the cell must partition the phosphorylated forms of CtrA and DivK into separate compartments. This can be achieved only if PleC in the swarmer compartment switches back to the phosphatase form and dephosphorylates DivK~P. In earlier work, we demonstrated that the phosphatase-to-kinase transitions are robust to small changes in the level of DivJ [57]. Compartmentalization creates a situation where DivJ is completely absent in the PleC-containing swarmer compartment. Devoid of its signal kinase, PleC shows a decline in kinase level while DivK gets dephosphorylated.

Our model is able to reproduce the defect in swarmer progeny development of Δ*pleC* and *pleC*_H610A_ mutants (Fig 8B). Given the absence of a functional phosphatase, DivK~P level remains high in the swarmer compartment of the mutant cells.

### Proposed molecular mechanism to produce different developmental fates in *Caulobacter*

**Fig 9:**
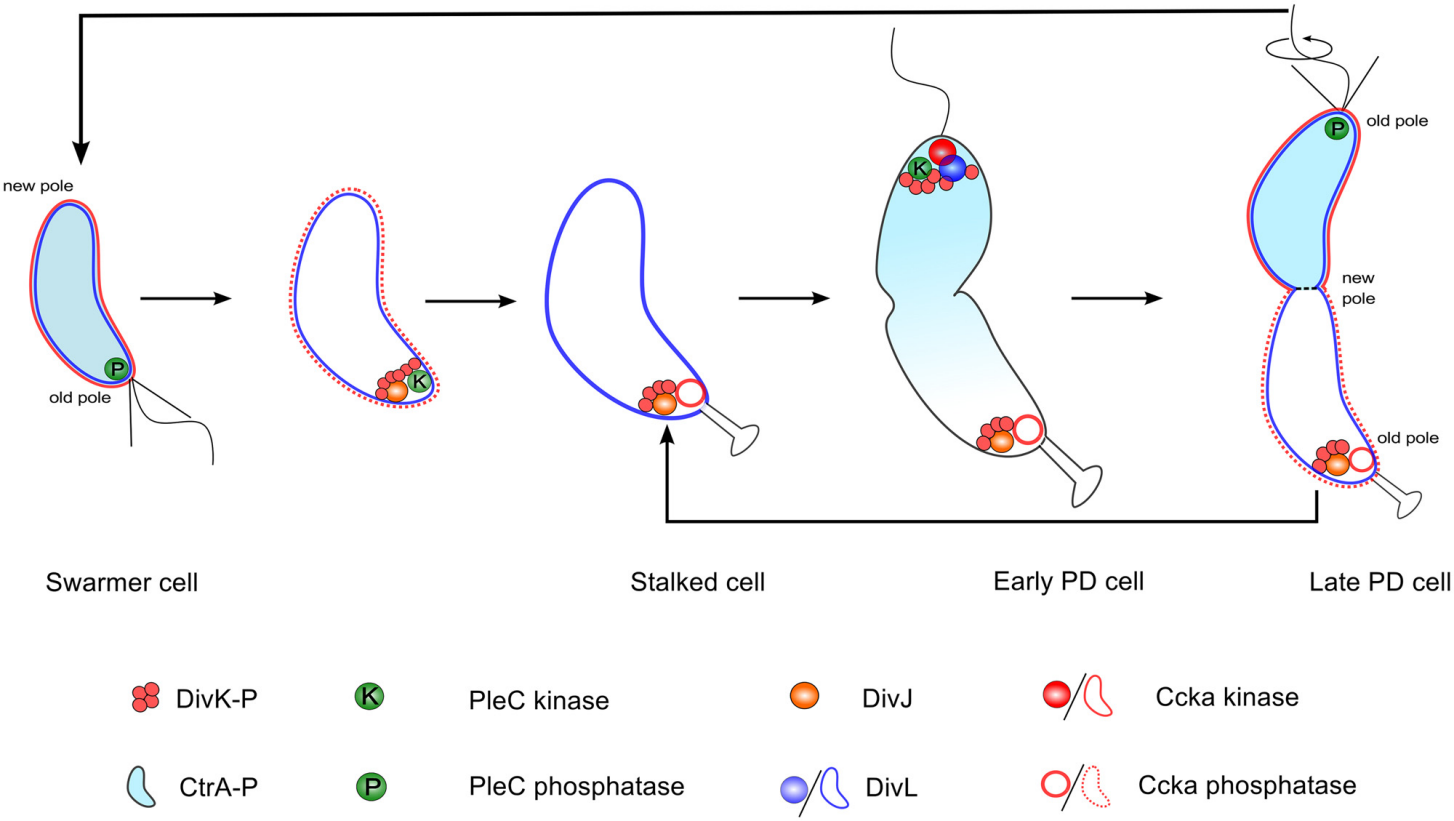
Localization and function of the DivK-PleC-DivK and DivL-CckA-CtrA signaling networks, as suggested by the model calculations reported here.

Fig 9 summarizes the localization and functional status of proteins, as proposed in our model. At the molecular level, the stalked cell is distinguished from the swarmer cell by the kinase DivJ, which localizes at the old pole and initiates the swarmer-to-stalked transition. Hence, DivJ can be considered as a cell-fate determinant protein [23]. However, there is no counterpart for DivJ that initiates the transition from stalked to predivisional cell. Instead, the cell recycles the same components—namely, DivJ-PleC-DivK and DivL-CckA-CtrA—to program the predivisional stage of the cell cycle.

Based on our simulation results, we make the case that rapid diffusion of DivK~P does not permit PleC to be a phosphatase prior to cytokinesis. The co-localization of DivL and PleC kinase at the new pole of the predivisional cell enables the effective sequestration of DivK~P by PleC kinase, thus allowing both DivK and CtrA to be phosphorylated at the same stage of the cell cycle. Here, we use our mathematical model to offer further insight into how *Caulobacter crescentus* exploits spatial localization to temporally regulate the activity of DivL, ultimately giving rise to cell fate asymmetry.

## Supporting Information

S1 Fig Spatiotemporal distribution of proteins when PleC is delocalized (at *t* = 30 min) from the old pole of the stalked cell. Color indicates concentration gradients from minimum (blue) to maximum (red). **(A)** DivJ is localized at the old pole (*t* = 30–120 min). PleC is delocalized from the old pole before it can transition to a kinase in the stalked cell. **(B)** Instead, PleC transitions to the kinase state in the new pole of the predivisional cell, enabling DivL to be in the active form. **(C)** CckA becoming a kinase, while old-pole CckA remains a phosphatase. Consequently, the predivisional cell establishes a gradient of CtrA~P along its length from high at the new pole to low at the old pole.
(TIF)

S2 Fig New-pole PleC is a phosphatase if *D*_DivK_ = *D*_DivK~P_ = 0.1 μm^2^ min^-1^. **(A)** PleC becomes a kinase at the old pole of the stalked cell (*t* = 30–70 minutes). Upon relocation to the new pole (*t* = 90 min), PleC reverts back to phosphatase form because of the slow diffusion of DivK~P. **(B)** Consequently, DivK is dephosphorylated in the new pole and fails to localize there, and new pole DivL is protected from the inhibitory effect of DivK~P. **(C)** CckA localized at the new pole is a kinase and the CtrA~P gradient is established in the predivisional cell.
(TIF)

S3 Fig The fraction of PleC kinase (green curve) and PleC phosphatase (red curve) that is bound to DivK and/or DivK~P is shown on a log-scale. (TIF)

S4 Fig Unipolar localization of DivK~P in *divK*_D90G_ strain is a consequence of PleC being a phosphatase. In the *divK*_D90G_ mutant, PleC does not transition to a kinase. Although some DivK_D90G_ is phosphorylated by DivJ at the old pole, DivK_D90G_~P is unable to up-regulate the kinase activity of PleC or to bind efficiently to DivL.
(TIF)

S1 Table Equations governing the model. (DOCX)

S2 Table Parameters for wild type simulations. (DOCX)

S3 Table Rate constants for mutant simulations*. (DOCX)

S4 Table Values of indicator functions *p*_*i*_. (DOCX)

S5 Table Summary of simulation results*. (DOCX)

## References

[1] Slaughter BD, Smith SE, Li R. Symmetry Breaking in the Life Cycle of the Budding Yeast. Cold Spring Harb Perspect Biol. 2009;1: 18 10.1101/cshperspect.a003384 PMC2773630 20066112

[2] Munro E, Bowerman B. Cellular symmetry breaking during Caenorhabditis elegans development. Cold Spring Harb Perspect Biol. 2009;1 10.1101/cshperspect.a003400 PMC2773627 20066102

[3] Petricka JJ, Van Norman JM, Benfey PN. Symmetry Breaking in Plants: Molecular Mechanisms Regulating Asymmetric Cell Divisions in Arabidopsis. 2009;1: 17 10.1101/cshperspect.a000497 PMC2773642 20066115

[4] Prehoda KE. Polarization of Drosophila neuroblasts during asymmetric division. Cold Spring Harb Perspect Biol. 2009;1: 1–13. 10.1101/cshperspect.a001388 PMC2742096 20066083

[5] Dworkin J. Cellular polarity in prokaryotic organisms. Cold Spring Harb Perspect Biol. 2009;1: 1–14. 10.1101/cshperspect.a003368 PMC2882128 20457568

[6] Winkler J, Seybert A, König L, Pruggnaller S, Haselmann U, Sourjik V, et al Quantitative and spatio-temporal features of protein aggregation in Escherichia coli and consequences on protein quality control and cellular ageing. EMBO J. 2010;29: 910–923. 10.1038/emboj.2009.412 20094032 PMC2837176

[7] Wu LJ, Errington J. RacA and the Soj-Spo0J system combine to effect polar chromosome segregation in sporulating Bacillus subtilis. Mol Microbiol. 2003;49: 1463–1475. 12950914 10.1046/j.1365-2958.2003.03643.x

[8] Dos Santos VT, Bisson-Filho AW, Gueiros-Filho FJ. DivIVA-Mediated Polar Localization of ComN, a Posttranscriptional Regulator of Bacillus subtilis. Journal of Bacteriology. 2012 pp. 3661–3669. 10.1128/JB.05879-11 22582279 PMC3393515

[9] Yamaichi Y, Bruckner R, Ringgaard S, Moll A, Cameron DE, Briegel A, et al A multidomain hub anchors the chromosome segregation and chemotactic machinery to the bacterial pole. Genes & Development. 2012 pp. 2348–2360. 23070816 10.1101/gad.199869.112 PMC3475806

[10] Goldberg MB, Bârzu O, Parsot C, Sansonetti PJ. Unipolar localization and ATPase activity of IcsA, a Shigella flexneri protein involved in intracellular movement. J Bacteriol. 1993;175: 2189–2196. 8468279 10.1128/jb.175.8.2189-2196.1993 PMC204503

[11] Steinhauer J, Agha R, Pham T, Varga AW, Goldberg MB. The unipolar Shigella surface protein IcsA is targeted directly to the bacterial old pole: IcsP cleavage of IcsA occurs over the entire bacterial surface. Mol Microbiol. 1999;32: 367–377. 10231492 10.1046/j.1365-2958.1999.01356.x

[12] Werner JN, Chen EY, Guberman JM, Zippilli AR, Irgon JJ, Gitai Z. Quantitative genome-scale analysis of protein localization in an asymmetric bacterium. Proc Natl Acad Sci U S A. 2009;106: 7858–7863. 10.1073/pnas.0901781106 19416866 PMC2671984

[13] Ausmees N, Jacobs-Wagner C. Spatial and temporal control of differentiation and cell cycle progression in Caulobacter crescentus. Annu Rev Microbiol. 2003;57: 225–247. Available: http://www.ncbi.nlm.nih.gov/pubmed/14527278 14527278 10.1146/annurev.micro.57.030502.091006

[14] Skerker JM, Laub MT. Cell-cycle progression and the generation of asymmetry in Caulobacter crescentus. Nat Rev Microbiol. 2004;2: 325–337. 10.1038/nrmicro864 15031731

[15] Curtis PD, Brun Y V. Getting in the Loop: Regulation of Development in Caulobacter crescentus. Microbiol Mol Biol Rev. 2010;74: 13–41. Available: http://mmbr.asm.org/cgi/content/abstract/74/1/13 10.1128/MMBR.00040-09 20197497 PMC2832345

[16] Aaron M, Charbon G, Lam H, Schwarz H, Vollmer W, Jacobs-Wagner C. The tubulin homologue FtsZ contributes to cell elongation by guiding cell wall precursor synthesis in Caulobacter crescentus. Mol Microbiol. 2007;64: 938–952. 10.1111/j.1365-2958.2007.05720.x 17501919

[17] Kühn J, Briegel A, Mörschel E, Kahnt J, Leser K, Wick S, et al Bactofilins, a ubiquitous class of cytoskeletal proteins mediating polar localization of a cell wall synthase in Caulobacter crescentus. EMBO J. 2010;29: 327–339. 10.1038/emboj.2009.358 19959992 PMC2824468

[18] Charbon G, Cabeen MT, Jacobs-Wagner C. Bacterial intermediate filaments: in vivo assembly, organization, and dynamics of crescentin. Genes Dev. 2009;23: 1131–1144. 10.1101/gad.1795509 19417107 PMC2682956

[19] Jin SK, Sun SX. Morphology of Caulobacter crescentus and the mechanical role of crescentin. Biophys J. 2009;96. 10.1016/j.bpj.2009.02.010 PMC2718293 19383443

[20] Lam H, Matroule J-Y, Jacobs-Wagner C. The asymmetric spatial distribution of bacterial signal transduction proteins coordinates cell cycle events. Dev Cell. 2003;5: 149–159. Available: http://www.ncbi.nlm.nih.gov/entrez/query.fcgi?cmd=Retrieve&db=PubMed&dopt=Citation&list_uids=12852859 12852859 10.1016/s1534-5807(03)00191-6

[21] Lam H, Schofield WB, Jacobs-Wagner C. A landmark protein essential for establishing and perpetuating the polarity of a bacterial cell. Cell. 2006;124: 1011–1023. 16530047 10.1016/j.cell.2005.12.040

[22] Briegel A, Ding HJ, Li Z, Werner J, Gitai Z, Dias DP, et al Location and architecture of the Caulobacter crescentus chemoreceptor array. Mol Microbiol. 2008;69: 30–41. 10.1111/j.1365-2958.2008.06219.x 18363791 PMC3090075

[23] Boutte CC, Henry JT, Crosson S. ppGpp and polyphosphate modulate cell cycle progression in Caulobacter crescentus. J Bacteriol. Am Soc Microbiol; 2012;194: 28–35. 10.1128/JB.05932-11 PMC3256613 22020649

[24] Henry JT, Crosson S. Chromosome replication and segregation govern the biogenesis and inheritance of inorganic polyphosphate granules. Mol Biol Cell. 2013;24: 3177–86. 10.1091/mbc.E13-04-0182 23985321 PMC3806658

[25] Goley ED, Yeh YC, Hong SH, Fero MJ, Abeliuk E, Mcadams HH, et al Assembly of the Caulobacter cell division machine. Mol Microbiol. 2011;80: 1680–1698. 10.1111/j.1365-2958.2011.07677.x 21542856 PMC3707389

[26] Hallez R, Bellefontaine A-F, Letesson J-J, De Bolle X. Morphological and functional asymmetry in alpha-proteobacteria. Trends Microbiol. 2004;12: 361–365. 10.1016/j.tim.2004.06.002 15276611

[27] Brilli M, Fondi M, Fani R, Mengoni A, Ferri L, Bazzicalupo M, et al The diversity and evolution of cell cycle regulation in alpha-proteobacteria: a comparative genomic analysis. BMC Syst Biol. 2010;4: 52 10.1186/1752-0509-4-52 20426835 PMC2877005

[28] Shapiro L, McAdams HH, Losick R. Generating and exploiting polarity in bacteria. Science. 2002;298: 1942–1946. 10.1126/science.1072163 12471245

[29] Kahng LS, Shapiro L. Polar localization of replicon origins in the multipartite genomes of Agrobacterium tumefaciens and Sinorhizobium meliloti. J Bacteriol. 2003;185: 3384–3391. 10.1128/JB.185.11.3384-3391.2003 12754237 PMC155372

[30] Hallez R, Mignolet J, Van Mullem V, Wery M, Vandenhaute J, Letesson J-J, et al The asymmetric distribution of the essential histidine kinase PdhS indicates a differentiation event in Brucella abortus. EMBO J. 2007;26: 1444–1455. 10.1038/sj.emboj.7601577 17304218 PMC1817626

[31] Shapiro L, McAdams HH, Losick R. Why and how bacteria localize proteins. Science. 2009;326: 1225–1228. 10.1126/science.1175685 19965466 PMC7531253

[32] Kobayashi H, De Nisco NJ, Chien P, Simmons LA, Walker GC. Sinorhizobium meliloti CpdR1 is critical for co-ordinating cell cycle progression and the symbiotic chronic infection. Mol Microbiol. 2009;73: 586–600. 10.1111/j.1365-2958.2009.06794.x 19602145 PMC2756024

[33] Laloux G, Jacobs-Wagner C. How do bacteria localize proteins to the cell pole? J Cell Sci. 2014;127: 11–9. 10.1242/jcs.138628 24345373 PMC3874780

[34] Stekhoven DJ, Omasits U, Quebatte M, Dehio C, Ahrens CH. Proteome-wide identification of predominant subcellular protein localizations in a bacterial model organism. J Proteomics. Elsevier B.V.; 2014;99: 123–137. 10.1016/j.jprot.2014.01.015 24486812

[35] Laub MT, Chen SL, Shapiro L, McAdams HH. Genes directly controlled by CtrA, a master regulator of the Caulobacter cell cycle. Proc Natl Acad Sci U S A. 2002;99: 4632–4637. Available: http://www.pubmedcentral.nih.gov/articlerender.fcgi?artid=123699&tool=pmcentrez&rendertype=abstract 11930012 10.1073/pnas.062065699 PMC123699

[36] Reisenauer A, Quon K, Shapiro L. The CtrA Response Regulator Mediates Temporal Control of Gene Expression during the Caulobacter Cell Cycle. J Bacteriol. 1999;181: 2430–2439. Available: http://www.pubmedcentral.nih.gov/articlerender.fcgi?artid=93667&tool=pmcentrez&rendertype=abstract 10198005 10.1128/jb.181.8.2430-2439.1999 PMC93667

[37] Reisenauer A, Shapiro L. DNA methylation affects the cell cycle transcription of the CtrA global regulator in Caulobacter. Eur Mol Biol Organ J. 2002;21: 4969–4977. Available: http://www.pubmedcentral.nih.gov/articlerender.fcgi?artid=126286&tool=pmcentrez&rendertype=abstract 10.1093/emboj/cdf490 PMC126286 12234936

[38] Collier J, McAdams HH, Shapiro L. A DNA methylation ratchet governs progression through a bacterial cell cycle. Proc Natl Acad Sci U S A. National Academy of Sciences.; 2007;104: 17111–17116. Available: http://www.pubmedcentral.nih.gov/articlerender.fcgi?artid=2040471&tool=pmcentrez&rendertype=abstract 10.1073/pnas.0708112104 PMC2040471 17942674

[39] Shen X, Collier J, Dill D, Shapiro L, Horowitz M, McAdams HH. Architecture and inherent robustness of a bacterial cell-cycle control system. Proc Natl Acad Sci U S A. 2008;105: 11340–11345. 10.1073/pnas.0805258105 18685108 PMC2516238

[40] Domian IJ, Reisenauer A, Shapiro L. Feedback control of a master bacterial cell-cycle regulator. Proc Natl Acad Sci U S A. The National Academy of Sciences.; 1999;96: 6648–6653. Available: http://www.pubmedcentral.nih.gov/articlerender.fcgi?artid=21969&tool=pmcentrez&rendertype=abstract 10.1073/pnas.96.12.6648 PMC21969 10359766

[41] Quon KC, Yang B, Domian IJ, Shapiro L, Marczynski GT. Negative control of bacterial DNA replication by a cell cycle regulatory protein that binds at the chromosome origin. Proc Natl Acad Sci U S A. National Academy of Sciences.; 1998;95: 120–125. Available: http://www.ncbi.nlm.nih.gov/cgi-bin/Entrez/referer?http://www.pnas.org/cgi/content/full/95/1/120 10.1073/pnas.95.1.120 PMC18146 9419339

[42] Paul R, Jaeger T, Abel S, Wiederkehr I, Folcher M, Biondi EG, et al Allosteric regulation of histidine kinases by their cognate response regulator determines cell fate. Cell. 2008;133: 452–461. Available: http://www.pubmedcentral.nih.gov/articlerender.fcgi?artid=2804905&tool=pmcentrez&rendertype=abstract 10.1016/j.cell.2008.02.045 18455986 PMC2804905

[43] Chen YE, Tsokos CG, Biondi EG, Perchuk BS, Laub MT. Dynamics of two Phosphorelays controlling cell cycle progression in Caulobacter crescentus. J Bacteriol. 2009;191: 7417–29. Available: http://www.pubmedcentral.nih.gov/articlerender.fcgi?artid=2786585&tool=pmcentrez&rendertype=abstract 10.1128/JB.00992-09 19783630 PMC2786585

[44] Reisinger SJ, Huntwork S, Viollier PH, Ryan KR. DivL Performs Critical Cell Cycle Functions in Caulobacter crescentus Independent of Kinase Activity. J Bacteriol. American Society for Microbiology (ASM); 2007;189: 8308–8320. Available: http://www.pubmedcentral.nih.gov/articlerender.fcgi?artid=2168681&tool=pmcentrez&rendertype=abstract 10.1128/JB.00868-07 PMC2168681 17827294

[45] Tsokos CG, Perchuk BS, Laub MT. A dynamic complex of signaling proteins uses polar localization to regulate cell fate asymmetry in Caulobacter crescentus. Dev Cell. 2011;20: 329–341. 10.1016/j.devcel.2011.01.007 21397844 PMC3068846

[46] Chen YE, Tropini C, Jonas K, Tsokos CG, Huang KC, Laub MT. Spatial gradient of protein phosphorylation underlies replicative asymmetry in a bacterium. Proc Natl Acad Sci U S A. The National Academy of Sciences.; 2011;108: 1052–1057. Available: http://www.pubmedcentral.nih.gov/articlerender.fcgi?artid=298695&tool=pmcentrez&rendertype=abstract 10.1073/pnas.1015397108 PMC3024676 21191097

[47] Siegal-Gaskins D, Crosson S. Tightly Regulated and Heritable Division Control in Single Bacterial Cells. Biophys J. The Biophysical Society.; 2008;95: 2063–2072. Available: http://www.pubmedcentral.nih.gov/articlerender.fcgi?artid=2483777&tool=pmcentrez&rendertype=abstract 10.1529/biophysj.108.128785 PMC2483777 18469083

[48] Radhakrishnan SK, Thanbichler M, Viollier PH. The dynamic interplay between a cell fate determinant and a lysozyme homolog drives the asymmetric division cycle of Caulobacter crescentus. Genes Dev. Cold Spring Harbor Laboratory Press.; 2008;22: 212–225. Available: http://www.pubmedcentral.nih.gov/articlerender.fcgi?artid=2192755&tool=pmcentrez&rendertype=abstract 10.1101/gad.1601808 PMC2192755 18198338

[49] Lin Y, Crosson S, Scherer NF. Single-gene tuning of Caulobacter cell cycle period and noise, swarming motility, and surface adhesion. Mol Syst Biol. Nature Publishing Group.; 2010;6: 445 Available: http://www.nature.com/doifinder/10.1038/msb.2010.95 10.1038/msb.2010.95 PMC3018171 21179017

[50] Biondi EG, Reisinger SJ, Skerker JM, Arif M, Perchuk BS, Ryan KR, et al Regulation of the bacterial cell cycle by an integrated genetic circuit. Nature. Nature Publishing Group.; 2006;444: 899–904. Available: http://www.ncbi.nlm.nih.gov/pubmed/17136100 10.1038/nature05321 17136100

[51] Jacobs C, Hung D, Shapiro L. Dynamic localization of a cytoplasmic signal transduction response regulator controls morphogenesis during the Caulobacter cell cycle. Proc Natl Acad Sci U S A. The National Academy of Sciences.; 2001;98: 4095–4100. Available: http://www.pubmedcentral.nih.gov/articlerender.fcgi?artid=31185&tool=pmcentrez&rendertype=abstract 10.1073/pnas.051609998 PMC31185 11274434

[52] Angelastro PS, Sliusarenko O, Jacobs-Wagner C. Polar localization of the CckA histidine kinase and cell cycle periodicity of the essential master regulator CtrA in Caulobacter crescentus. J Bacteriol. American Society for Microbiology (ASM); 2010;192: 539–552. Available: http://www.pubmedcentral.nih.gov/articlerender.fcgi?artid=2805319&tool=pmcentrez&rendertype=abstract 10.1128/JB.00985-09 PMC2805319 19897656

[53] Matroule J-Y, Lam H, Burnette DT, Jacobs-Wagner C. Cytokinesis monitoring during development; rapid pole-to-pole shuttling of a signaling protein by localized kinase and phosphatase in Caulobacter. Cell. 2004;118: 579–590. Available: http://www.ncbi.nlm.nih.gov/pubmed/15339663 15339663 10.1016/j.cell.2004.08.019

[54] Jenal U, Galperin MY. Single domain response regulators: molecular switches with emerging roles in cell organization and dynamics. Current Opinion in Microbiology. 2009 pp. 152–160. 10.1016/j.mib.2009.01.010 19246239 PMC2725762

[55] Thanbichler M. Spatial regulation in Caulobacter crescentus. Current Opinion in Microbiology. 2009 pp. 715–721. 10.1016/j.mib.2009.09.013 19854671

[56] Thanbichler M. Synchronization of chromosome dynamics and cell division in bacteria. Cold Spring Harb Perspect Biol. 2010;2: a000331 10.1101/cshperspect.a000331 20182599 PMC2827906

[57] Subramanian K, Paul MR, Tyson JJ. Potential Role of a Bistable Histidine Kinase Switch in the Asymmetric Division Cycle of Caulobacter crescentus. PLoS Comput Biol. 2013;9. 10.1371/journal.pcbi.1003221 PMC3772055 24068904

[58] Wheeler RT, Shapiro L. Differential localization of two histidine kinases controlling bacterial cell differentiation. Mol Cell. Elsevier; 1999;4: 683–694. Available: http://www.ncbi.nlm.nih.gov/pubmed/10619016 10.1016/s1097-2765(00)80379-2 10619016

[59] Chen JC, Viollier PH, Shapiro L. A membrane metalloprotease participates in the sequential degradation of a Caulobacter polarity determinant. Mol Microbiol. 2005;55: 1085–1103. Available: http://www.ncbi.nlm.nih.gov/pubmed/15686556 15686556 10.1111/j.1365-2958.2004.04443.x

[60] Curtis PD, Quardokus EM, Lawler ML, Guo X, Klein D, Chen JC, et al The scaffolding and signalling functions of a localization factor impact polar development. Mol Microbiol. 2012;84: 1–24. 10.1111/j.1365-2958.2012.08055.x 22512778 PMC3345042

[61] Iniesta AA, Hillson NJ, Shapiro L. Cell pole-specific activation of a critical bacterial cell cycle kinase. Proc Natl Acad Sci U S A. National Academy of Sciences.; 2010;107: 7012–7017. Available: http://www.pubmedcentral.nih.gov/articlerender.fcgi?artid=2872457&tool=pmcentrez&rendertype=abstract 10.1073/pnas.1001767107 PMC2872457 20351295

[62] Iniesta AA, Hillson NJ, Shapiro L. Polar Remodeling and Histidine Kinase Activation, Which Is Essential for Caulobacter Cell Cycle Progression, Are Dependent on DNA Replication Initiation. J Bacteriol. American Society for Microbiology (ASM).; 2010;192: 3893–3902. Available: http://www.pubmedcentral.nih.gov/articlerender.fcgi?artid=2916389&tool=pmcentrez&rendertype=abstract 10.1128/JB.00468-10 PMC2916389 20525830

[63] Shampine LF, Reichelt MW. The MATLAB ODE Suite. SIAM Journal on Scientific Computing. 1997 pp. 1–22. 10.1137/S1064827594276424

[64] Hunter JD. Matplotlib: A 2D Graphics Environment. Comput Sci Eng. IEEE COMPUTER SOC.; 2007;9: 90–95. 10.1109/MCSE.2007.55

[65] Aldridge P, Paul R, Goymer P, Rainey P, Jenal U. Role of the GGDEF regulator PleD in polar development of Caulobacter crescentus. Mol Microbiol. 2003;47: 1695–1708. Available: http://www.ncbi.nlm.nih.gov/pubmed/12622822 12622822 10.1046/j.1365-2958.2003.03401.x

[66] Jonas K, Chen YE, Laub MT. Modularity of the bacterial cell cycle enables independent spatial and temporal control of DNA replication. Curr Biol. 2011;21: 1092–1101. 10.1016/j.cub.2011.05.040 21683595 PMC3143580

[67] Christen B, Fero MJ, Hillson NJ, Bowman G, Hong S-H, Shapiro L, et al High-throughput identification of protein localization dependency networks. Proc Natl Acad Sci U S A. 2010;107: 4681–4686. 10.1073/pnas.1000846107 20176934 PMC2842071

[68] Campos M, Surovtsev IV, Kato S, Paintdakhi A, Beltran B, Ebmeier SE, et al A Constant Size Extension Drives Bacterial Cell Size Homeostasis. Cell. Elsevier Inc.; 2014;159: 1433–1446. 10.1016/j.cell.2014.11.022 PMC4258233 25480302

[69] Ebersbach G, Briegel A, Jensen GJ, Jacobs-Wagner C. A self-associating protein critical for chromosome attachment, division, and polar organization in caulobacter. Cell. 2008;134: 956–968. Available: http://www.pubmedcentral.nih.gov/articlerender.fcgi?artid=2614312&tool=pmcentrez&rendertype=abstract 10.1016/j.cell.2008.07.016 18805089 PMC2614312

[70] Jacobs C, Domian IJ, Maddock JR, Shapiro L. Cell cycle-dependent polar localization of an essential bacterial histidine kinase that controls DNA replication and cell division. Cell. 1999;97: 111–20. Available: http://www.ncbi.nlm.nih.gov/pubmed/10199407 10199407 10.1016/s0092-8674(00)80719-9

[71] Spencer W, Siam R, Ouimet M-C, Bastedo DP, Marczynski GT. CtrA, a global response regulator, uses a distinct second category of weak DNA binding sites for cell cycle transcription control in Caulobacter crescentus. J Bacteriol. American Society for Microbiology (ASM).; 2009;191: 5458–5470. Available: http://www.pubmedcentral.nih.gov/articlerender.fcgi?artid=2725627&tool=pmcentrez&rendertype=abstract 10.1128/JB.00355-09 PMC2725627 19542275

[72] Hung DY, Shapiro L. A signal transduction protein cues proteolytic events critical to Caulobacter cell cycle progression. Proc Natl Acad Sci U S A. The National Academy of Sciences.; 2002;99: 13160–13165. Available: http://www.pubmedcentral.nih.gov/articlerender.fcgi?artid=130603&tool=pmcentrez&rendertype=abstract 10.1073/pnas.202495099 PMC130603 12237413

[73] Iniesta AA, Shapiro L. A bacterial control circuit integrates polar localization and proteolysis of key regulatory proteins with a phospho-signaling cascade. Proc Natl Acad Sci U S A. 2008;105: 16602–7. Available: http://www.pubmedcentral.nih.gov/articlerender.fcgi?artid=2575466&tool=pmcentrez&rendertype=abstract 10.1073/pnas.0808807105 18946044 PMC2575466

[74] Gorbatyuk B, Marczynski GT. Regulated degradation of chromosome replication proteins DnaA and CtrA in Caulobacter crescentus. Mol Microbiol. 2005;55: 1233–1245. Available: http://www.ncbi.nlm.nih.gov/pubmed/15686567 15686567 10.1111/j.1365-2958.2004.04459.x

[75] Tropini C, Huang KC. Interplay between the Localization and Kinetics of Phosphorylation in Flagellar Pole Development of the Bacterium Caulobacter crescentus. PLoS Comput Biol. 2012;8: e1002602 10.1371/journal.pcbi.1002602 22876167 PMC3410866

[76] Li S, Brazhnik P, Sobral B, Tyson JJ. A Quantitative Study of the Division Cycle of Caulobacter crescentus Stalked Cells. Arkin AP, editor. PLoS Comput Biol. Public Library of Science.; 2008;4: 19 Available: http://www.pubmedcentral.nih.gov/articlerender.fcgi?artid=2217572&tool=pmcentrez&rendertype=abstract 10.1371/journal.pcbi.0040009 PMC2217572 18225942

[77] Li S, Brazhnik P, Sobral B, Tyson JJ. Temporal Controls of the Asymmetric Cell Division Cycle in Caulobacter crescentus. Papin JA, editor. PLoS Comput Biol. Public Library of Science; 2009;5: 15 Available: http://www.ncbi.nlm.nih.gov/pubmed/19680425 10.1371/journal.pcbi.1000463 PMC2714070 19680425

[78] Ryan KR, Judd EM, Shapiro L. The CtrA response regulator essential for Caulobacter crescentus cell-cycle progression requires a bipartite degradation signal for temporally controlled proteolysis. J Mol Biol. 2002;324: 443–455. 10.1016/S0022-2836(02)01042-2 12445780

[79] Iniesta AA, McGrath PT, Reisenauer A, McAdams HH, Shapiro L. A phospho-signaling pathway controls the localization and activity of a protease complex critical for bacterial cell cycle progression. Proc Natl Acad Sci U S A. National Academy of Sciences.; 2006;103: 10935–10940. Available: http://www.pubmedcentral.nih.gov/articlerender.fcgi?artid=1544152&tool=pmcentrez&rendertype=abstract 10.1073/pnas.0604554103 PMC1544152 16829582

[80] Abel S, Chien P, Wassmann P, Schirmer T, Kaever V, Laub MT, et al Regulatory Cohesion of Cell Cycle and Cell Differentiation through Interlinked Phosphorylation and Second Messenger Networks. Mol Cell. 2011;43: 550–560. 10.1016/j.molcel.2011.07.018 21855795 PMC3298681

[81] Domian IJ, Quon KC, Shapiro L. Cell type-specific phosphorylation and proteolysis of a transcriptional regulator controls the G1-to-S transition in a bacterial cell cycle. Cell. 1997;90: 415–424. Available: http://www.ncbi.nlm.nih.gov/pubmed/9150145 9267022 10.1016/s0092-8674(00)80502-4

