# Supplementary figures and images for: Dynamical Localization of DivL and PleC in the Asymmetric Division Cycle of Caulobacter crescentus: A Theoretical Investigation of Alternative Models
Source: PLoS Comput Biol. 2015 Jul 17;11(7):e1004348. doi: 10.1371/journal.pcbi.1004348 (PMC4505887; doi:10.1371/journal.pcbi.1004348)

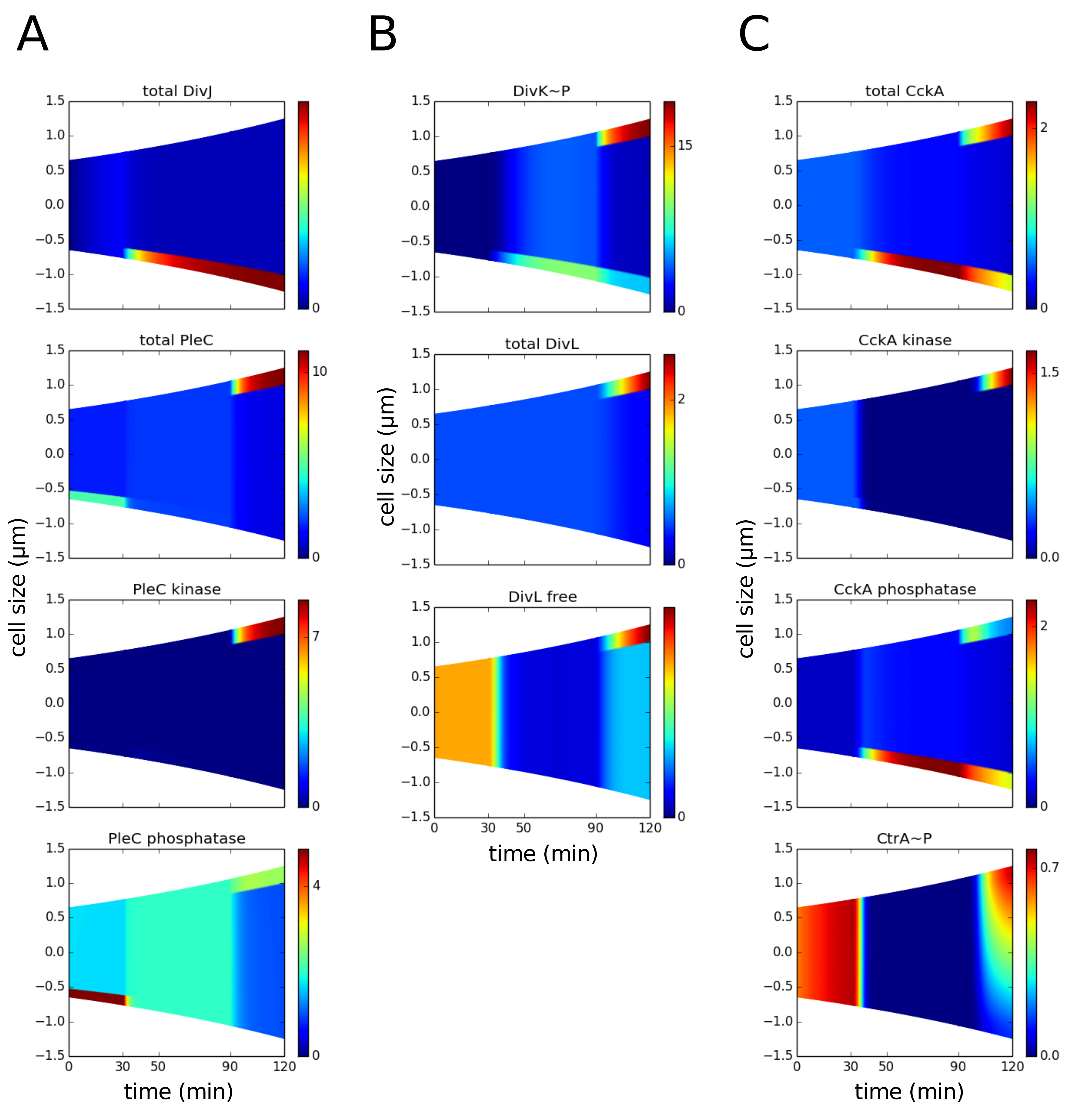

Supplement: S1 Fig — Color indicates concentration gradients from minimum (blue) to maximum (red). (A) DivJ is localized at the old pole (t = 30–120 min). PleC is delocalized from the old pole before it can transition to a kinase in the stalked cell. (B) Instead, PleC transitions to the kinase state in the new pole of the predivisional cell, enabling DivL to be in the active form. (C) CckA becoming a kinase, while old-pole CckA remains a phosphatase. Consequently, the predivisional cell establishes a gradient of CtrA~P along its length from high at the new pole to low at the old pole. (TIF) [file pcbi.1004348.s001.tif]

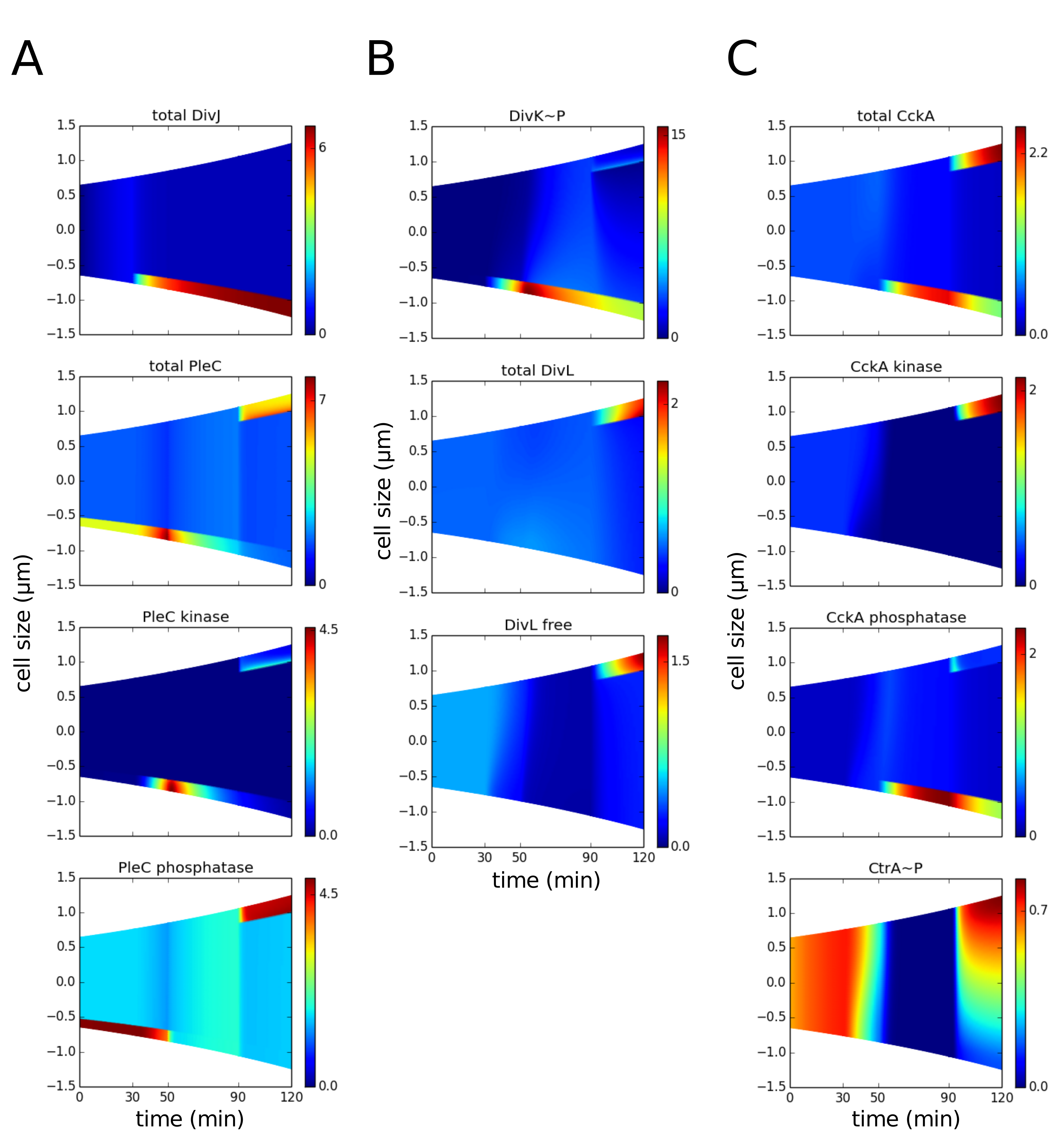

Supplement: S2 Fig — (A) PleC becomes a kinase at the old pole of the stalked cell (t = 30–70 minutes). Upon relocation to the new pole (t = 90 min), PleC reverts back to phosphatase form because of the slow diffusion of DivK~P. (B) Consequently, DivK is dephosphorylated in the new pole and fails to localize there, and new pole DivL is protected from the inhibitory effect of DivK~P. (C) CckA localized at the new pole is a kinase and the CtrA~P gradient is established in the predivisional cell. (TIF) [file pcbi.1004348.s002.tif]

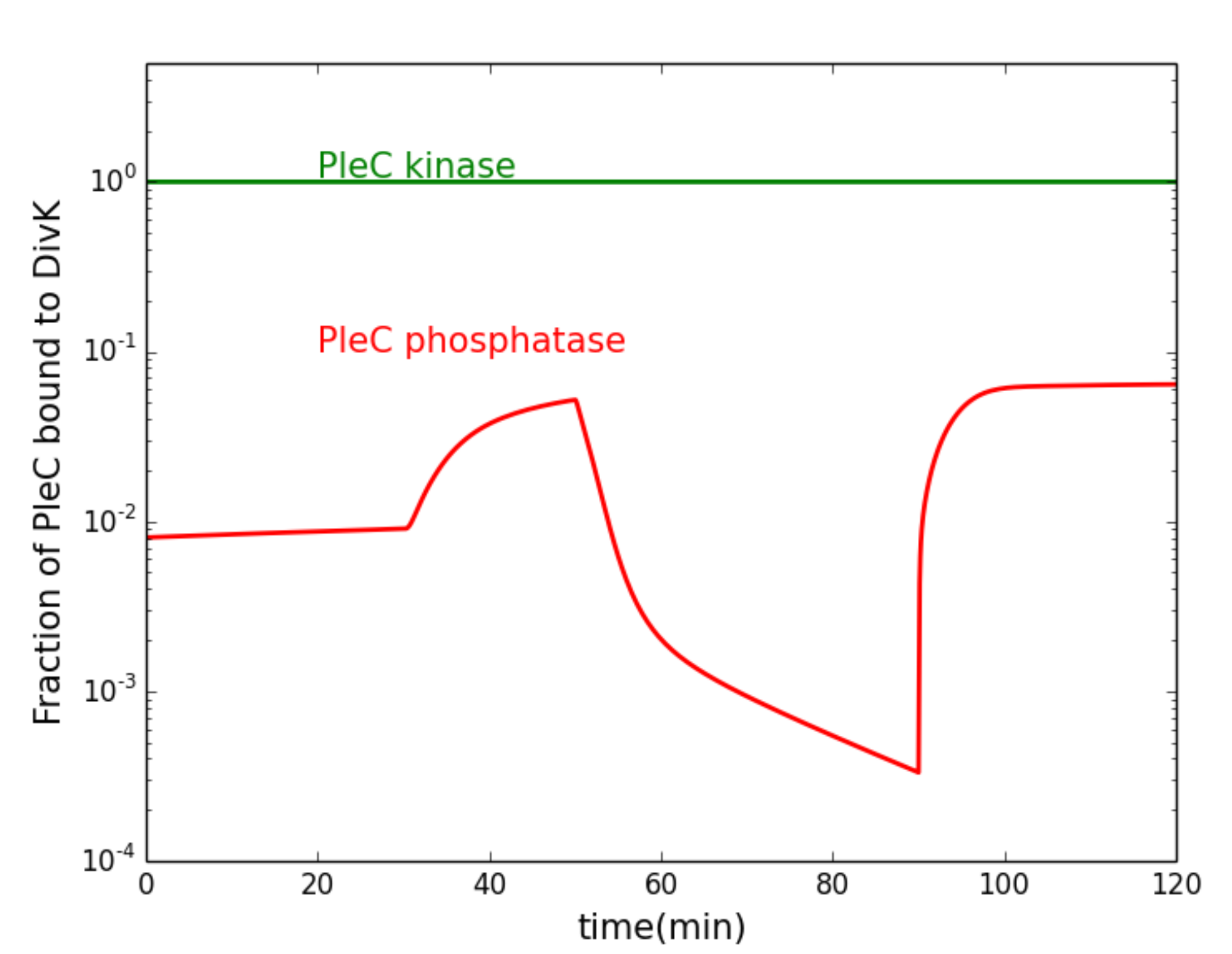

Supplement: S3 Fig — (TIF) [file pcbi.1004348.s003.tif]

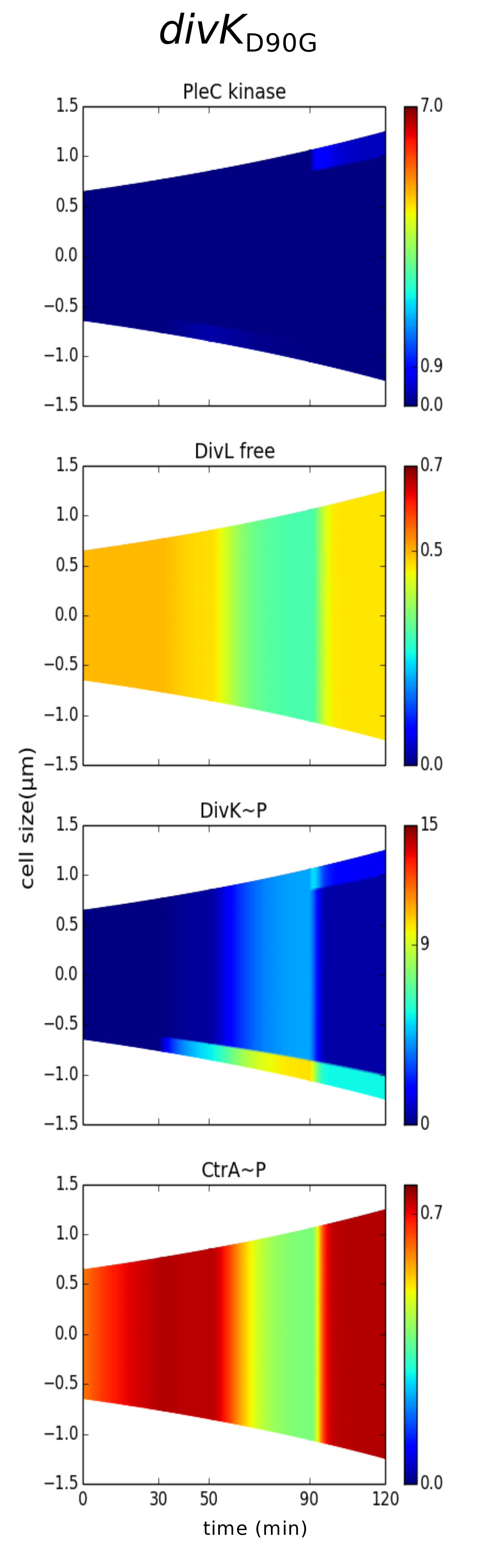

Supplement: S4 Fig — In the divK D90G mutant, PleC does not transition to a kinase. Although some DivKD90G is phosphorylated by DivJ at the old pole, DivKD90G~P is unable to up-regulate the kinase activity of PleC or to bind efficiently to DivL. (TIF) [file pcbi.1004348.s004.tif]
