# Supplementary material for: Dynamical Localization of DivL and PleC in the Asymmetric Division Cycle of Caulobacter crescentus: A Theoretical Investigation of Alternative Models
Source: PLoS Comput Biol. 2015 Jul 17;11(7):e1004348. doi: 10.1371/journal.pcbi.1004348 (PMC4505887; doi:10.1371/journal.pcbi.1004348)
Supplement: S1 Table — (DOCX) [file pcbi.1004348.s005.docx]

Table S1: Equations governing the model

| $\frac{\partial\left[ DivK\sim P \right]}{\partial t} = -\left( k_{deg-dkp}+k_{\mathrm{dil}} \right)\cdot\left[ DivK\sim P \right]- k_{pc-ph1}\cdot\left[ \mathrm{PleC} \right]\cdot\left[ DivK\sim P \right] +k_{ph1-pc}\cdot\left[ \mathrm{Ple}C_{h1} \right]-k_{ph1-pk11}\cdot\left[ \mathrm{Ple}C_{ph1} \right]\cdot\left[ DivK\sim P \right] +k_{pk11-ph1}\cdot\left[ \mathrm{PleC}_{kin11} \right] -k_{ph2-pk12}\cdot\left[ \mathrm{Ple}C_{ph2} \right]\cdot\left[ DivK\sim P \right] + k_{pk12-ph2}\cdot\left[ \mathrm{Ple}C_{kin12} \right]-k_{pk1-pk0}\cdot\left[ \mathrm{Ple}C_{kin1} \right]\cdot\left[ DivK\sim P \right] +k_{pk0-pk1}\cdot\left[ \mathrm{Ple}C_{kin0} \right]- k_{pk3-pk2}\cdot\left[ \mathrm{Ple}C_{kin3} \right]\cdot\left[ DivK\sim P \right] + k_{pk2-pk3}\cdot\left[ \mathrm{Ple}C_{kin2} \right] + k_{pt2-pk1h}\cdot\left[ \mathrm{Ple}C_{pt2} \right] -k_{pk1h-pt2}\cdot\left[ \mathrm{Ple}C_{kin1h} \right]\cdot\left[ DivK\sim P \right]-k_{pk2p-pk1} \cdot\left[ \mathrm{Ple}C_{kin2p} \right]\cdot\left[ DivK\sim P \right]+ k_{pk1-pk2p}\cdot\left[ \mathrm{Ple}C_{kin1} \right]- k_{pk1p-pk1h}\cdot\left[ \mathrm{Ple}C_{kin1p} \right]\cdot\left[ DivK\sim P \right] + k_{pk1h-pk1p}\cdot\left[ \mathrm{Ple}C_{kin1h} \right] +k_{pt3-pk1p} \cdot\left[ \mathrm{Ple}C_{pt3} \right] - k_{pk1p-pt3}\cdot\left[ \mathrm{Ple}C_{kin1p} \right]\cdot\left[ DivK\sim P \right] -k_{j-jkp}\cdot\left[ \mathrm{DivJ}_{b} \right]\cdot\left[ DivK\sim P \right] + k_{jkp-j}\cdot\left[ \mathrm{DivJ}_{\text{b}}:DivK\sim P \right] - k_{pk3h-pt4}\cdot\left[ \mathrm{PleC}_{kin3h} \right]\cdot\left[ DivK\sim P \right] + k_{pt4-pk3h}\cdot\left[ \mathrm{PleC}_{pt4} \right]+ k_{h1-h2}\cdot\left[ \mathrm{Ple}C_{ph1} \right]\cdot\left[ \mathrm{DivK} \right] - k_{h2-h1}\cdot\left[ \mathrm{Ple}C_{ph2} \right]\cdot\left[ DivK\sim P \right] +D_{\mathrm{DivKP}}\cdot\frac{\partial^{2}\left[ DivK\sim P \right]}{\partial x^{2}}$ | [1] |
| --- | --- |
| $\frac{\partial\left[ \mathrm{DivK} \right]}{\partial t} = k_{syn-dk}-{(k}_{deg-dk}+k_{\mathrm{dil}})\cdot\left[ \mathrm{DivK} \right]- k_{pc-ph2}\cdot\left[ \mathrm{PleC} \right]\cdot\left[ \mathrm{DivK} \right]+k_{ph2-pc}\cdot\left[ \mathrm{Ple}C_{h2} \right]- k_{ph2-pk22}\cdot\left[ \mathrm{Ple}C_{ph2} \right]\cdot\left[ \mathrm{DivK} \right]+ k_{pk22-ph2}\cdot\left[ \mathrm{PleC}_{kin22} \right] -k_{ph1-pk12}\cdot\left[ \mathrm{Ple}C_{ph1} \right]\cdot\left[ \mathrm{DivK} \right]+ k_{pk12-ph1}\cdot\left[ \mathrm{Ple}C_{kin12} \right] -k_{pk1-pk2}\cdot\left[ \mathrm{Ple}C_{kin1} \right]\cdot\left[ \mathrm{DivK} \right]+k_{pk2-pk1}\cdot\left[ \mathrm{Ple}C_{kin2} \right] - k_{pk3-pk4}\cdot\left[ \mathrm{Ple}C_{kin3} \right]\cdot\left[ \mathrm{DivK} \right]+ k_{pk4-pk3}\cdot\left[ \mathrm{Ple}C_{kin4} \right] - k_{pk2p-pk3}\cdot\left[ \mathrm{Ple}C_{pk2p} \right]\cdot\left[ \mathrm{DivK} \right]+k_{pk3-pk2p}\cdot\left[ \mathrm{Ple}C_{kin3} \right] -k_{j-jk}\cdot\left[ \mathrm{DivJ}_{\text{b}} \right]\cdot\left[ \mathrm{DivK} \right]+ k_{jk-j}\cdot\left[ \mathrm{DivJ}_{\text{b}}:DivK \right]- k_{ph1-pk12}\cdot\left[ \mathrm{Ple}C_{h1} \right]\cdot\left[ \mathrm{DivK} \right]+ k_{pk12-ph1}\cdot\left[ \mathrm{Ple}C_{kin12} \right]- k_{pk1p-pk3h}\cdot\left[ \mathrm{Ple}C_{kin1p} \right]\cdot\left[ \mathrm{DivK} \right] +k_{pk3h-pk1p}\cdot\left[ \mathrm{Ple}C_{kin3h} \right]- k_{h1-h2}\cdot\left[ \mathrm{Ple}C_{ph1} \right]\cdot\left[ \mathrm{DivK} \right] + k_{h2-h1}\cdot\left[ \mathrm{Ple}C_{ph2} \right]\cdot\left[ DivK\sim P \right] +D_{\mathrm{DivK}}\cdot\frac{\partial^{2}\left[ \mathrm{DivK} \right]}{\partial x^{2}}$ | [2] |
| $\frac{\partial\left[ \mathrm{PleC}_{f} \right]}{\partial t} = k_{syn\_plc}-{(k}_{deg-plc}+k_{\mathrm{dil}})\cdot\left[ \mathrm{PleC}_{f} \right]- k_{pcf-pcb}\cdot p_{\mathrm{plc}}(x)\cdot\left[ \mathrm{PleC}_{f} \right] +k_{pcb-pcf}\cdot\left[ \mathrm{Ple}C_{b} \right]+D_{\mathrm{PleC}}\cdot\frac{\partial^{2}\left[ \mathrm{PleC}_{f} \right]}{\partial x^{2}}$ | [3] |
| $\frac{d\left[ \mathrm{PleC}_{b} \right]}{dt} = k_{pcf-pcb}\cdot p_{\mathrm{plc}}\left( x \right)\cdot\left[ \mathrm{PleC}_{f} \right]-k_{pcb-pcf}\cdot\left[ \mathrm{Ple}C_{b} \right]- k_{pc-ph2}\cdot\left[ \mathrm{PleC}_{b} \right]\cdot\left[ \mathrm{DivK} \right] +k_{ph2-pc}\cdot\left[ \mathrm{Ple}C_{h2} \right] - k_{pc-ph1}\cdot\left[ \mathrm{PleC}_{b} \right]\cdot\left[ DivK\sim P \right]+k_{ph1-pc}\cdot\left[ \mathrm{Ple}C_{h1} \right]+k_{ph2p-pc}\cdot\left[ \mathrm{Ple}C_{h2p} \right]+k_{ph1p-pc}\cdot\left[ \mathrm{Ple}C_{h1p} \right]-{(k}_{deg-plc}+k_{\mathrm{dil}})\cdot\left[ \mathrm{PleC}_{b} \right]$ | [4] |
| $\frac{d\left[ \mathrm{PleC}_{ph1} \right]}{dt} = k_{pc-ph1}\cdot\left[ \mathrm{PleC}_{b} \right]\cdot\left[ DivK\sim P \right]-k_{ph1-pc}\cdot\left[ \mathrm{Ple}C_{h1} \right] - k_{ph1-pk11}\cdot\left[ \mathrm{Ple}C_{ph1} \right]\cdot\left[ DivK\sim P \right]+ k_{pk11-ph1}\cdot\left[ \mathrm{PleC}_{kin11} \right] - k_{ph1-pk12}\cdot\left[ \mathrm{Ple}C_{ph1} \right]\cdot\left[ \mathrm{DivK} \right] + k_{pk12-ph1}\cdot\left[ \mathrm{Ple}C_{kin12} \right] -k_{h1-h2}\cdot\left[ \mathrm{Ple}C_{ph1} \right]\cdot\left[ \mathrm{DivK} \right]+k_{h2-h1}\cdot\left[ \mathrm{Ple}C_{ph2} \right]\cdot\left[ DivK\sim P \right] -k_{ph1-ph2}\cdot\left[ \mathrm{Ple}C_{ph1} \right] +k_{ph2-ph1}\cdot\left[ \mathrm{Ple}C_{ph2} \right]-{(k}_{deg-plc}+k_{\mathrm{dil}})\cdot\left[ \mathrm{PleC}_{ph1} \right]$ | [5] |
| $\frac{d\left[ \mathrm{PleC}_{ph2} \right]}{dt} = k_{pc-ph2}\cdot\left[ \mathrm{PleC}_{b} \right]\cdot\left[ \mathrm{DivK} \right]-k_{ph2-pc}\cdot\left[ \mathrm{Ple}C_{ph2} \right] - k_{ph2-pk22}\cdot\left[ \mathrm{Ple}C_{ph2} \right]\cdot\left[ \mathrm{DivK} \right]+ k_{pk22-ph2}\cdot\left[ \mathrm{PleC}_{kin22} \right] -k_{ph2-pk12}\cdot\left[ \mathrm{Ple}C_{ph2} \right]\cdot\left[ DivK\sim P \right] + k_{pk12-ph2}\cdot\left[ \mathrm{Ple}C_{kin12} \right]+k_{h1-h2}\cdot\left[ \mathrm{Ple}C_{ph1} \right]\cdot\left[ \mathrm{DivK} \right] -k_{h2-h1}\cdot\left[ \mathrm{Ple}C_{ph2} \right]\cdot\left[ DivK\sim P \right] +k_{ph1-ph2}\cdot\left[ \mathrm{Ple}C_{ph1} \right]-k_{ph2-ph1}\cdot\left[ \mathrm{Ple}C_{ph2} \right]-{(k}_{deg-plc}+k_{\mathrm{dil}})\cdot\left[ \mathrm{PleC}_{ph2} \right]$ | [6] |
| ${\frac{d\left[ \mathrm{PleC}_{kin11} \right]}{dt} =k_{ph1-p11}\cdot\left[ \mathrm{Ple}C_{ph1} \right]\cdot\left[ DivK\sim P \right]-k_{pk11-ph1}\cdot\left[ \mathrm{PleC}_{kin11} \right] - k_{pk11-pk0}\cdot\left[ \mathrm{PleC}_{kin11} \right]+ k_{pk0-pk11}\cdot\left[ \mathrm{PleC}_{kin0} \right]+ k}_{pt4-pk11}\cdot\left[ \mathrm{PleC}_{pt4} \right]- k_{pk11-pt4}\cdot\left[ \mathrm{PleC}_{kin11} \right]-{(k}_{deg-plc}+k_{\mathrm{dil}})\cdot\left[ \mathrm{PleC}_{kin11} \right]$ | [7] |
| $\frac{d\left[ \mathrm{PleC}_{kin12} \right]}{dt} =k_{ph2-pk12}\cdot\left[ \mathrm{Ple}C_{ph2} \right]\cdot\left[ DivK\sim P \right]- k_{pk12-ph2}\cdot\left[ \mathrm{Ple}C_{kin12} \right] + k_{ph1-pk12}\cdot\left[ \mathrm{Ple}C_{ph1} \right]\cdot\left[ \mathrm{DivK} \right]- k_{pk12-ph1}\cdot\left[ \mathrm{Ple}C_{kin12} \right] - k_{pk22-pk2}\cdot\left[ \mathrm{PleC}_{kin22} \right]+ k_{pk2-pk22}\cdot\left[ \mathrm{PleC}_{kin2} \right] -{(k}_{deg-plc}+k_{\mathrm{dil}})\cdot\left[ \mathrm{PleC}_{kin12} \right]$ | [8] |
| ${\frac{d\left[ \mathrm{PleC}_{kin22} \right]}{dt} =k_{ph2-pk22}\cdot\left[ \mathrm{Ple}C_{h2} \right]\cdot\left[ \mathrm{DivK} \right]- k_{pk22-ph2}\cdot\left[ \mathrm{PleC}_{kin22} \right]}- k_{p22-pk4}\cdot\left[ \mathrm{PleC}_{kin22} \right]+ k_{pk4-pk22}\cdot\left[ \mathrm{PleC}_{kin4} \right]-{(k}_{deg-plc}+k_{\mathrm{dil}})\cdot\left[ \mathrm{PleC}_{kin22} \right]$ | [9] |
| $\frac{d\left[ \mathrm{PleC}_{kin0} \right]}{dt} = k_{pk11-pk0}\cdot\left[ \mathrm{PleC}_{kin11} \right]- k_{pk0-pk11}\cdot\left[ \mathrm{PleC}_{kin0} \right] - k_{pk0-pk1}\cdot\left[ \mathrm{PleC}_{kin0} \right]+ k_{pk1-pk0}\cdot\left[ \mathrm{PleC}_{kin1} \right]\cdot\left[ DivK\sim P \right]-{(k}_{deg-plc}+k_{\mathrm{dil}})\cdot\left[ \mathrm{PleC}_{kin0} \right]$ | [10] |
| $\frac{d\left[ \mathrm{PleC}_{kin2} \right]}{dt} = k_{pk12-pk2}\cdot\left[ \mathrm{PleC}_{kin12} \right]-k_{pk2-pk11}\cdot\left[ \mathrm{PleC}_{kin2} \right]- k_{pk2-pk1}\cdot\left[ \mathrm{PleC}_{kin2} \right]+k_{pk1-pk2}\cdot\left[ \mathrm{PleC}_{kin1} \right]\cdot\left[ \mathrm{DivK} \right]- k_{pk2-pk3}\cdot\left[ \mathrm{PleC}_{kin2} \right] + k_{pk3-pk2}\cdot\left[ \mathrm{PleC}_{kin3} \right]\cdot\left[ DivK\sim P \right]+ k_{pt2-pk2}\cdot\left[ \mathrm{PleC}_{pt2} \right] - k_{pk2-pt2}\cdot\left[ \mathrm{PleC}_{kin2} \right]-{(k}_{deg-plc}+k_{\mathrm{dil}})\cdot\left[ \mathrm{PleC}_{kin2} \right]$ | [11] |
| $\frac{d\left[ \mathrm{PleC}_{kin4} \right]}{dt} = k_{pk22-pk4}\cdot\left[ \mathrm{PleC}_{kin22} \right]-k_{pk4-pk22}\cdot\left[ \mathrm{PleC}_{kin4} \right]-k_{pk4-pk3}\cdot\left[ \mathrm{PleC}_{kin4} \right] + k_{pk3-pk4}\cdot\left[ \mathrm{PleC}_{kin3} \right]\cdot\left[ \mathrm{DivK} \right] {+ k}_{pt4-pk4}\cdot\left[ \mathrm{PleC}_{pt4} \right]- k_{pk4-pt4}\cdot\left[ \mathrm{PleC}_{kin4} \right]-{(k}_{deg-plc}+k_{\mathrm{dil}})\cdot\left[ \mathrm{PleC}_{kin4} \right]$ | [12] |
| $\frac{d\left[ \mathrm{PleC}_{kin1} \right]}{dt} = -k_{pk1-pk0}\cdot\left[ \mathrm{Ple}C_{kin1} \right]\cdot\left[ DivK\sim P \right]+k_{pk0-pk1}\cdot\left[ \mathrm{Ple}C_{kin0} \right] -k_{pk1-pk2}\cdot\left[ \mathrm{Ple}C_{kin1} \right]\cdot\left[ \mathrm{DivK} \right]+k_{pk2-pk1}\cdot\left[ \mathrm{Ple}C_{kin2} \right]{+ k}_{pk2p-pk1} \cdot\left[ \mathrm{Ple}C_{kin2p} \right]\cdot\left[ DivK\sim P \right]- k_{pk1-pk2p}\cdot\left[ \mathrm{Ple}C_{kin1} \right]+ k_{pk1h-pk1}\cdot\left[ \mathrm{Ple}C_{kin1h} \right]- k_{pk1-pk1h}\cdot\left[ \mathrm{Ple}C_{kin1} \right]- k_{pk1-pk5}\cdot\left[ \mathrm{Ple}C_{kin1} \right]\cdot\left[ \mathrm{PleD} \right] + k_{pk5-pk1}\cdot\left[ \mathrm{Ple}C_{kin5} \right] -{(k}_{deg-plc}+k_{\mathrm{dil}})\cdot\left[ \mathrm{PleC}_{kin1} \right]$ | [13] |
| $\frac{d\left[ \mathrm{PleC}_{kin3} \right]}{dt} = -k_{pk3-pk2}\cdot\left[ \mathrm{Ple}C_{kin3} \right]\cdot\left[ DivK\sim P \right]+k_{pk2-pk3}\cdot\left[ \mathrm{Ple}C_{kin2} \right] -k_{pk3-pk4}\cdot\left[ \mathrm{Ple}C_{kin3} \right]\cdot\left[ \mathrm{DivK} \right]+k_{pk4-pk3}\cdot\left[ \mathrm{Ple}C_{kin4} \right]{+ k}_{pk2p-pk3} \cdot\left[ \mathrm{Ple}C_{kin2p} \right]\cdot\left[ \mathrm{DivK} \right]- k_{pk3-pk2p}\cdot\left[ \mathrm{Ple}C_{kin3} \right]+ k_{pk3h-pk3}\cdot\left[ \mathrm{Ple}C_{kin3h} \right]-k_{pk3-pk3h}\cdot\left[ \mathrm{Ple}C_{kin3} \right]+ k_{pt3-pk3}\cdot\left[ \mathrm{PleC}_{pt3} \right]- k_{pk3-pt3}\cdot\left[ \mathrm{PleC}_{kin3} \right]- k_{pk3-pk6}\cdot\left[ \mathrm{Ple}C_{kin3} \right]\cdot\left[ \mathrm{PleD} \right]+ k_{pk6-pk3}\cdot\left[ \mathrm{Ple}C_{kin6} \right]-{(k}_{deg-plc}+k_{\mathrm{dil}})\cdot\left[ \mathrm{PleC}_{kin3} \right]$ | [14] |
| $\frac{d\left[ \mathrm{PleC}_{pt2} \right]}{dt} = k_{pk1h-pt2}\cdot\left[ \mathrm{PleC}_{kin1h} \right]\cdot\left[ DivK\sim P \right]- k_{pt2-pk1h}\cdot\left[ \mathrm{PleC}_{pt2} \right]- k_{pt2-pk2}\cdot\left[ \mathrm{PleC}_{pt2} \right]+ k_{pk2-pt2}\cdot\left[ \mathrm{PleC}_{kin2} \right]-{(k}_{deg-plc}+k_{\mathrm{dil}})\cdot\left[ \mathrm{PleC}_{pt2} \right]$ | [15] |
| $\frac{d\left[ \mathrm{PleC}_{pt3} \right]}{dt} = k_{pk1p-pt3}\cdot\left[ \mathrm{PleC}_{kin1p} \right]\cdot\left[ DivK\sim P \right]- k_{pt3-pk1p}\cdot\left[ \mathrm{PleC}_{pt3} \right]- k_{pt3-pk3}\cdot\left[ \mathrm{PleC}_{pt3} \right]+ k_{pk3-pt3}\cdot\left[ \mathrm{PleC}_{kin3} \right]-{(k}_{deg-plc}+k_{\mathrm{dil}})\cdot\left[ \mathrm{PleC}_{pt3} \right]$ | [16] |
| $\frac{d\left[ \mathrm{PleC}_{pt4} \right]}{dt} = k_{pk3h-pt4}\cdot\left[ \mathrm{PleC}_{kin3h} \right]\cdot\left[ DivK\sim P \right]- k_{pt4-pk3h}\cdot\left[ \mathrm{PleC}_{pt4} \right]- k_{pt4-pk4}\cdot\left[ \mathrm{PleC}_{pt4} \right]+ k_{pk4-pt4}\cdot\left[ \mathrm{PleC}_{kin4} \right] - k_{pt4-p11}\cdot\left[ \mathrm{PleC}_{pt4} \right]+ k_{p11-pt4}\cdot\left[ \mathrm{PleC}_{kin11} \right]-{(k}_{deg-plc}+k_{\mathrm{dil}})\cdot\left[ \mathrm{PleC}_{pt4} \right]$ | [17] |
| $\frac{d\left[ \mathrm{PleC}_{kin1h} \right]}{dt} = - k_{pk1h-pt2}\cdot\left[ \mathrm{PleC}_{kin1h} \right]\cdot\left[ DivK\sim P \right]+ k_{pt2-pk1h}\cdot\left[ \mathrm{PleC}_{pt2} \right] -k_{pk1h-pk1}\cdot\left[ \mathrm{Ple}C_{kin1h} \right]+ k_{pk1-pk1h}\cdot\left[ \mathrm{Ple}C_{kin1} \right] + k_{pk1p-pk1h}\cdot\left[ \mathrm{Ple}C_{kin1p} \right]\cdot\left[ DivK\sim P \right]- k_{pk1h-pk1p}\cdot\left[ \mathrm{Ple}C_{kin1h} \right] -{(k}_{deg-plc}+k_{\mathrm{dil}})\cdot\left[ \mathrm{PleC}_{kin1h} \right]$ | [18] |
| $\frac{d\left[ \mathrm{PleC}_{kin3h} \right]}{dt} = -k_{pk3h-pk3}\cdot\left[ \mathrm{Ple}C_{kin3h} \right]+ k_{pk3-pk3h}\cdot\left[ \mathrm{Ple}C_{kin3} \right] + k_{pk1p-pk3h}\cdot\left[ \mathrm{Ple}C_{kin1p} \right]\cdot\left[ \mathrm{DivK} \right]- k_{pk3h-pk1p}\cdot\left[ \mathrm{Ple}C_{kin3h} \right] - k_{pk3h-pt4}\cdot\left[ \mathrm{PleC}_{kin3h} \right]\cdot\left[ DivK\sim P \right]+ k_{pt4-pk3h}\cdot\left[ \mathrm{PleC}_{pt4} \right] -{(k}_{deg-plc}+k_{\mathrm{dil}})\cdot\left[ \mathrm{PleC}_{kin3h} \right]$ | [19] |
| $\frac{d\left[ \mathrm{PleC}_{kin2p} \right]}{dt} = -k_{pk2p-pk1} \cdot\left[ \mathrm{Ple}C_{kin2p} \right]\cdot\left[ DivK\sim P \right]+ k_{pk1-pk2p}\cdot\left[ \mathrm{Ple}C_{kin1} \right] - k_{pk2p-pk3}\cdot\left[ \mathrm{Ple}C_{pk2p} \right]\cdot\left[ \mathrm{DivK} \right]+k_{pk3-pk2p}\cdot\left[ \mathrm{Ple}C_{kin3} \right] - k_{pk2p-pc}\cdot\left[ \mathrm{Ple}C_{kin2p} \right]-{(k}_{deg-plc}+k_{\mathrm{dil}})\cdot\left[ \mathrm{PleC}_{kin2p} \right]$ | [20] |
| $\frac{d\left[ \mathrm{PleC}_{kin1p} \right]}{dt} = -k_{pk1p-pk1h} \cdot\left[ \mathrm{Ple}C_{kin1p} \right]\cdot\left[ DivK\sim P \right]+ k_{pk1h-pk1p}\cdot\left[ \mathrm{Ple}C_{kin1h} \right] - k_{pk1p-pt3}\cdot\left[ \mathrm{Ple}C_{pk1p} \right]\cdot\left[ \mathrm{DivK} \right]+k_{pt3-pk1p}\cdot\left[ \mathrm{Ple}C_{pt3} \right] - k_{pk1p-pc}\cdot\left[ \mathrm{Ple}C_{kin1p} \right]- k_{pk1p-pk3h}\cdot\left[ \mathrm{Ple}C_{kin1p} \right]\cdot\left[ \mathrm{DivK} \right] + k_{pk3h-pk1p}\cdot\left[ \mathrm{Ple}C_{kin3h} \right]-{(k}_{deg-plc}+k_{\mathrm{dil}})\cdot\left[ \mathrm{PleC}_{kin1p} \right]$ | [21] |
| $\frac{\partial\left[ \mathrm{DivJ}_{f} \right]}{\partial t} = k_{syn\_dj}-{(k}_{deg-dj}+k_{\mathrm{dil}})\cdot\left[ \mathrm{DivJ}_{f} \right]- k_{djf-djb}\cdot p_{\mathrm{dj}}(x)\cdot\left[ \mathrm{DivJ}_{f} \right]+k_{djb-djf}\cdot\left[ \mathrm{DivJ}_{b} \right] +D_{\mathrm{DivJ}}\cdot\frac{\partial^{2}\left[ \mathrm{DivJ}_{f} \right]}{\partial x^{2}}$ | [22] |
| $\frac{d\left[ \mathrm{DivJ}_{b} \right]}{dt} = k_{djf-djb}\cdot p_{\mathrm{dj}}\left( x \right)\cdot\left[ \mathrm{DivJ}_{f} \right]-k_{djb-djf}\cdot\left[ \mathrm{DivJ}_{b} \right]-k_{j-jk}\cdot\left[ \mathrm{DivJ}_{b} \right]\cdot\left[ \mathrm{DivK} \right] + k_{jk-j}\cdot\left[ \mathrm{DivJ}_{\text{b}}:DivK \right]-k_{j-jkp}\cdot\left[ \mathrm{DivJ}_{b} \right]\cdot\left[ DivK\sim P \right]+ k_{jkp-j}\cdot\left[ \mathrm{DivJ}_{\text{b}}:DivK\sim P \right]-{(k}_{deg-dj}+k_{\mathrm{dil}})\cdot\left[ \mathrm{DivJ}_{b} \right]$ | [23] |
| $\frac{d\left[ \mathrm{DivJ}_{\text{b}}:DivK \right]}{dt} = k_{j-jk}\cdot\left[ \mathrm{DivJ}_{\text{b}} \right]\cdot\left[ \mathrm{DivK} \right]- k_{jk-j}\cdot\left[ \mathrm{DivJ}_{\text{b}}:DivK \right] - k_{jk-jkp}\cdot\left[ \mathrm{DivJ}_{\text{b}}:DivK \right]{+ k}_{jkp-jk}\cdot\left[ \mathrm{DivJ}_{\text{b}}:DivK\sim P \right] -{(k}_{deg-dj}+k_{\mathrm{dil}})\cdot\left[ \mathrm{DivJ}_{\text{b}}:DivK \right]$ | [24] |
| $\frac{d\left[ \mathrm{DivJ}_{\text{b}}:DivK\sim P \right]}{dt} = k_{jk-jkp}\cdot\left[ \mathrm{DivJ}_{\text{b}}:DivK \right]{- k}_{jkp-jk}\cdot\left[ \mathrm{DivJ}_{\text{b}}:DivK\sim P \right]+k_{j-jkp}\cdot\left[ \mathrm{DivJ}_{b} \right]\cdot\left[ DivK\sim P \right]- k_{jkp-j}\cdot\left[ \mathrm{DivJ}_{\text{b}}:DivK\sim P \right]-{(k}_{deg-dj}+k_{\mathrm{dil}})\cdot\left[ \mathrm{DivJ}_{\text{b}}:DivK\sim P \right]$ | [25] |
| $\frac{\partial\left[ \mathrm{DivL}_{f} \right]}{\partial t} = k_{syn\_dl}-{(k}_{\deg}+k_{\mathrm{dil}})\cdot\left[ \mathrm{DivL}_{f} \right]- k_{dlf-dlb}\cdot p_{\mathrm{dl}}(x)\cdot\left[ \mathrm{DivL}_{f} \right] +k_{dlb-dlf}\cdot\left[ \mathrm{DivL}_{b} \right]+D_{\mathrm{DivL}}\cdot\frac{\partial^{2}\left[ \mathrm{DivL}_{f} \right]}{\partial x^{2}}$ | [26] |
| $\frac{d\left[ \mathrm{DivL}_{b} \right]}{dt} = k_{dlf-dlb}\cdot p_{\mathrm{dl}}(x)\cdot\left[ \mathrm{DivL}_{f} \right]-k_{dlb-dlf}\cdot\left[ \mathrm{DivL}_{b} \right]-k_{dl-dldk}\cdot\left[ \mathrm{DivL}_{b} \right]\cdot\left[ DivK\sim P \right]+ k_{dldk-dl}\cdot\left[ \mathrm{DivL}_{b}:DivK\sim P \right]-{(k}_{\deg}+k_{\mathrm{dil}})\cdot\left[ \mathrm{DivL}_{b} \right]$ | [27] |
| $\frac{d\left[ \mathrm{DivL}_{b}:DivK\sim P \right]}{dt}= k_{dl-dldk}\cdot\left[ \mathrm{DivL}_{b} \right]\cdot\left[ DivK\sim P \right]- k_{dldk-dl}\cdot\left[ \mathrm{DivL}_{b}:DivK\sim P \right] -{(k}_{\deg}+k_{\mathrm{dil}})\cdot\left[ \mathrm{DivL}_{b}:DivK\sim P \right]$ | [28] |
| $\frac{\partial\left[ \mathrm{CtrA} \right]}{\partial t} = k_{syn-ctr}- {(k}_{deg-ctr}+k_{\mathrm{dil}})\cdot\left[ \mathrm{CtrA} \right]- k_{\mathrm{ctr}_{\mathrm{kin}}}\cdot\left[ \mathrm{CckA}_{\mathrm{kin}} \right]\cdot\left[ \mathrm{CtrA} \right] +k_{ctr\_phos}\cdot\left[ \mathrm{CckA}_{\mathrm{phos}} \right]\cdot\left[ CtrA\sim P \right]+D_{\mathrm{CtrA}}\cdot\frac{\partial^{2}\left[ \mathrm{CtrA} \right]}{\partial x^{2}}$ | [29] |
| $\frac{\partial\left[ CtrA\sim P \right]}{\partial t} = - {(k}_{deg-ctr}+k_{\mathrm{dil}})\cdot\left[ CtrA\sim P \right]+ k_{\mathrm{ctr}_{\mathrm{kin}}}\cdot\left[ \mathrm{CckA}_{\mathrm{kin}} \right]\cdot\left[ \mathrm{CtrA} \right] -k_{ctr\_phos}\cdot\left[ \mathrm{CckA}_{\mathrm{phos}} \right]\cdot\left[ CtrA\sim P \right]+D_{\mathrm{CtrAP}}\cdot\frac{\partial^{2}\left[ CtrA\sim P \right]}{\partial x^{2}}$ | [30] |
| $\frac{\partial\left[ \mathrm{CckA}_{f} \right]}{\partial t}= k_{syn\_ccka}-k_{\deg}\cdot\left[ \mathrm{CckA}_{f} \right]- k_{ckf-ckb}\cdot p_{\mathrm{ccka}}(x)\cdot\left[ \mathrm{CckA}_{f} \right]+k_{ckb-ckf}\cdot\left[ \mathrm{CckA}_{\mathrm{phos}} \right]+D_{\mathrm{CckA}}\cdot\frac{\partial^{2}\left[ \mathrm{CckA}_{f} \right]}{\partial x^{2}}$ | [31] |
| $\frac{d\left[ \mathrm{CckA}_{\mathrm{phos}} \right]}{dt} = k_{ckf-ckb}\cdot p_{\mathrm{ccka}}\left( x \right)\cdot\left[ \mathrm{CckA}_{f} \right]-k_{ckb-ckf}\cdot\left[ \mathrm{CckA}_{\mathrm{phos}} \right] -k_{cp-ck}\cdot\left[ \mathrm{CckA}_{\mathrm{phos}} \right]\cdot\frac{\left[ \mathrm{DivL}_{b} \right]^{4}}{K_{m-dl}^{4}+ \left[ \mathrm{DivL}_{b} \right]^{4}} +k_{ck-cp}\cdot\left[ \mathrm{CckA}_{\mathrm{kin}} \right] -{(k}_{\deg}+k_{\mathrm{dil}})\cdot\left[ \mathrm{CckA}_{\mathrm{phos}} \right]$ | [32] |
| $\frac{d\left[ \mathrm{CckA}_{\mathrm{kin}} \right]}{dt} = k_{cp-ck}\cdot\left[ \mathrm{CckA}_{\mathrm{phos}} \right]\cdot\frac{\left[ \mathrm{DivL}_{b} \right]^{4}}{K_{m-dl}^{4} + \left[ \mathrm{DivL}_{b} \right]^{4}}-k_{ck-cp}\cdot\left[ \mathrm{CckA}_{\mathrm{kin}} \right] -{(k}_{\deg}+k_{\mathrm{dil}})\cdot\left[ \mathrm{CckA}_{\mathrm{kin}} \right]$ | [33] |
