# Supplementary material for: Dynamical Localization of DivL and PleC in the Asymmetric Division Cycle of Caulobacter crescentus: A Theoretical Investigation of Alternative Models
Source: PLoS Comput Biol. 2015 Jul 17;11(7):e1004348. doi: 10.1371/journal.pcbi.1004348 (PMC4505887; doi:10.1371/journal.pcbi.1004348)
Supplement: S2 Table — (DOCX) [file pcbi.1004348.s006.docx]

Table S2: Parameters for wild type simulations

| *Synthesis and degradation rate constants* (min^-1^) | | | | PleC | *k*_pk1p_1h_ = 5 | *k*_1h_pk1p_ = 0.16 | |
| --- | --- | --- | --- | --- | --- | --- | --- |
| DivJ | *k*_syn_dj_ = 0.05 | | *k*_deg_dj_ = 0.05 |  | *k*_pk3_pk2p_=0.0016 | *k*_pk2p_pk3_ = 5 | |
| DivK | *k*_syn_dk_ = 0.05 | | *k*_deg_dk_ = 0.005 |  | *k*_pk3_pk4_ = 5 | *k*_pk4_pk3_ = 0.0016 | |
|  | *k*_deg_dkp_ = 0.005 | |  |  | *k*_pk4_pt4_ = 5 | *k*_pt4_pk4_ = 0.16 | |
| PleC | *k*_syn_plc_ = 0.1 | | *k*_deg_plc_ = 0.05 |  | *k*_pc_ph2_ = 0.05 | *k*_ph2_pc_ = 5 | |
|  | *k*_deg_dkp_ = 0.005 | |  |  | *k*_ph2_p22_ = 0.016 | *k*_p22_ph22_ = 1.6$\cdot10^{-8}$ | |
| DivL | *k*_syn_dl_ = 0.025 | | *k*_deg_dl_ = 0.05 |  | *k*_p22_pk4_ = 5 | *k*_pk4_p22_ = 5 | |
| CckA | *k*_syn_ccka_ = 0.025 | | *k*_deg_ccka_ = 0.05 |  | *k*_pt4_pk3h_ = 0.16 | *k*_pk3h_pt4_ = 5 | |
| CtrA | *k*_syn_ctr_ = 0.05 | | *k*_deg_ctr_ = 0.05 |  | *k*_pk3_pk3h_ = 5 | *k*_pk3h_pk3_ = 5 | |
|  | | | |  | *k*_pk1p_p3h_ = 5 | *k*_p3h_pk1p_ = 0.0016 | |
| *Polar binding rate constants* (min^-1^) | | | |  | *k*_ph1_p12_= 0.0016 | *k*_p12_ph1_ = 1.6$\cdot10^{-4}$ | |
| DivJ | *k*_djf_djb_ = 1 | *k*_djb_djf_ = 0 | |  | *k*_p12_pk2_ = 5 | *k*_pk2_p12_ = 5 | |
| PleC | *k*_pcf_pcb_ = 1 | *k*_pcb_pcf_ = 0.5 | |  | *k*_ph2_p12_ = 1.6 | *k*_p12_ph2_ = 1.6$\cdot10^{-4}$ | |
| DivL | *k*_dlf_dlb_ = 1 | *k*_dlb_clf_ = 0.1 | |  | *k*_h1_h2_ = 0.0016 | *k*_h2_h1_ = 1.6 | |
| CckA | *k*_ckf_ckb_ = 1 | *k*_ckb_ckf_ = 0.1 | |  | *k*_p11_pt4_ = 0.0075 | *k*_pt4_p11_ = 5 | |
|  |  |  | |  | *k*_ph1_ph2_ = 10 | *k*_ph2_ph1_ = 0.005 | |
| *Phosphatase-kinase transition, autophosphorylat- ion & phosphotransfer rate constants* (min^-1^) | | | |  | *k*_pt3_pk1p_ = 0.16 | *k*_pk1p_pt3_ = 5 | |
|  |  |  |  |  |  |  | |
| DivJ | *k*_j_jk_ = 5 | *k*_jk_j_ = 0.0016 | | CckA | *k*_cp_ck_ = 10 | *k*_ck_cp_ = 1 | |
|  | *k*_jk_jkp_ = 5 | *k*_jkp_jk_ = 0.16 | |  |  |  | |
|  | *k*_jkp_dj_= 1 | *k*_dj_jkp_ = 5 | | CtrA | *k*_ctr_kin_ = 600 | *k*_ctr_phos_ = 600 | |
|  |  |  | | DivL | *k*_bdl_dldk_ = 2.5 | *k*_dldk_dl_ = 0.5 | |
| PleC | *k*_pc_ph1_ = 5 | *k*_ph1_pc_ = 5 | |  |  |  | |
|  | *k*_ph1_p11_ = 5 | *k*_pk11_ph1_ = 2.5 | | *Diffusion rate constants* (μm^2^ min^-1^) | | | |
|  | *k*_p11_pk0_ = 2.5 | *k*_pk0_p11_ = 5 | |  | *D*_DivK_= 100 | | *D*_DivKP_ = 100 |
|  | *k*_pk0_pk1_ = 0.16 | *k*_pk1_pk0_ = 5 | |  | *D*_DivJ_ = 100 | | *D*_DivL_ = 100 |
|  | *k*_pk1_pk2_ = 5 | *k*_pk2_pk1_ = 0.0016 | |  | *D*_PleC_ = 10 | | *D*_CckA_ = 100 |
|  | *k*_pk1_pk1h_ = 5 | *k*_pk1h_pk1_ = 5 | |  | *D*_CtrA_ = 100 | | *D*_CtrAP_ = 100 |
|  | *k*_pk1_pk2p_ = 0.16 | *k*_pk2p_pk1_ = 5 | |  |  | |  |
|  | *k*_pk2_pt2_ = 5 | *k*_pt2_pk2_ = 0.16 | | *Growth rate constant* (min^-1^) | | | |
|  | *k*_pt2_pk1h_ = 0.16 | *k*_pk1h_pt2_ = 5 | |  | *k*_growth_ = 0.0055 | | |
|  | *k*_pk2p_pc_ = 5 | *k*_pk1p_pc_ = 5 | |  |  | |  |
|  | *k*_pk2_pk3_ = 0.16 | *k*_pk3_pk2_ = 5 | | Equilibrium constant (dimensionless) | | | |
|  | *k*_pk3_pt3_ = 5 | *k*_pt3_pk3_ = 0.16 | |  | *K*_mdl_ = 0.5 | |  |
