# Supplementary material for: Dynamical Localization of DivL and PleC in the Asymmetric Division Cycle of Caulobacter crescentus: A Theoretical Investigation of Alternative Models
Source: PLoS Comput Biol. 2015 Jul 17;11(7):e1004348. doi: 10.1371/journal.pcbi.1004348 (PMC4505887; doi:10.1371/journal.pcbi.1004348)
Supplement: S3 Table — (DOCX) [file pcbi.1004348.s007.docx]

Table S3: Rate constants for mutant simulations*

| Δ*pleC* | *k*_syn_plc_ = 0 |  | *pleC*_H610A_ | *k*_p11_pk0_ = 0 | *k*_pk0_p11_ = 0 |
| --- | --- | --- | --- | --- | --- |
|  |  |  |  | *k*_p22_pk4_ = 0 | *k*_pk4_p22_ = 0 |
| *pleC*_F778L_ | *k*_p11_pk0_ = 0 | *k*_pk0_p11_ = 0 |  | *k*_p12_pk2_ = 0 | *k*_pk2_p12_ = 0 |
|  | *k*_p22_pk4_ = 0 | *k*_pk4_p22_ = 0 |  | *k*_pk2_pt2_ = 0 | *k*_pt2_pk2_ = 0 |
|  | *k*_p12_pk2_ = 0 | *k*_pk2_p12_ = 0 |  | *k*_pk3_pt3_ = 0 | *k*_pt3_pk3_ = 0 |
|  | *k*_pk2_pt2_ = 0 | *k*_pt2_pk2_ = 0 |  | *k*_pk4_pt4_ = 0 | *k*_pt4_pk4_ = 0 |
|  | *k*_pk3_pt3_ = 0 | *k*_pt3_pk3_ = 0 |  | *k*_p11_pt4_ = 0 | *k*_pt4_p11_ = 0 |
|  | *k*_pk4_pt4_ = 0 | *k*_pt4_pk4_ = 0 |  | *k*_ph1_ph2_ = 0 | *k*_ph2_ph1_ = 0 |
|  | *k*_p11_pt4_ = 0 | *k*_pt4_p11_ = 0 |  |  |  |
|  |  |  | *divK*_D90G_ | *k*_bdl_dldk_ = 0.1 | *k*_udl_dldk_ = 0.5 |
| Δ*divJ* | *k*_syn_dk_ = 0 |  |  | *k*_ph1_p11_ = 2.5 | *k*_p11_ph1_ = 50 |
|  |  |  |  | *k*_ph2_p22_ = 0.025 | *k*_p22_ph2_ = 0.002 |
| *divK*_D53N_ | *k*_jk_jkp_ = 0 | *k*_jkp_jk_ = 0 |  | *k*_ph1_p12_ = 0.016 | *k*_p12_ph1_ = 16 |
|  | *k*_pk2_pt2_ = 0 | *k*_pt2_pk2_ = 0 |  | *k*_ph2_p12_ = 1.6 | *k*_p12_ph2_ = 16 |
|  | *k*_pk3_pt3_ = 0 | *k*_pt3_pk3_ = 0 |  |  |  |
|  | *k*_pk4_pt4_ = 0 | *k*_pt4_pk4_ = 0 | *divJ*_H338A_ | *k*_jk_jkp_ = 0 | *k*_jkp_jk_ = 0 |
|  | *k*_p11_pt4_ = 0 | *k*_pt4_p11_ = 0 |  |  |  |
|  |  |  |  |  |  |
| DivK overexpression | *k*_syn_dk_ = 0.2 (4×) | *k*_syn_dk_ = 0.4 (8×) |  |  |  |
|  |  |  |  |  |  |
| * All rate constants have units of min^-1^ | | | | | |
