# Supplementary material for: Dynamical Localization of DivL and PleC in the Asymmetric Division Cycle of Caulobacter crescentus: A Theoretical Investigation of Alternative Models
Source: PLoS Comput Biol. 2015 Jul 17;11(7):e1004348. doi: 10.1371/journal.pcbi.1004348 (PMC4505887; doi:10.1371/journal.pcbi.1004348)
Supplement: S4 Table — (DOCX) [file pcbi.1004348.s008.docx]

Table S4: Values of indicator functions *p_i_*

|  | *t* = 0 – 30 min | *t* = 30 – 50 min | *t* = 50 – 90 min | *t* = 90 – 120 min | *t* = 120 – 150 min |
| --- | --- | --- | --- | --- | --- |
| DivJ | $p_{i}^{dj}=0$ * | $p_{i}^{dj}$= 1 for 1 ≤ *i* ≤ 10 (old pole) and $p_{i}^{dj}$= 0 for *i* > 10 | | | |
| PleC | $p_{i}^{plc}$= 1 for 1 ≤ *i* ≤ 10 (old pole) and $p_{i}^{plc}$= 0 for *i* > 10 | | $p_{i}^{plc}=0$* | $p_{i}^{plc}$= 1 for 91 ≤ *i* ≤ 100 (new pole) and $p_{i}^{plc}$= 0 for *i* < 91 | |
| DivL | $p_{i}^{dl}=1$* | | | $p_{i}^{dl}$= 1 for 91 ≤ *i* ≤ 100 (new pole) and $p_{i}^{dl}$= 0 for *i* < 91 | |
| CckA | $p_{i}^{ccka}=1$* | $p_{i}^{ccka}$= 1 for 1 ≤ *i* ≤ 10 (old pole) and $p_{i}^{ccka}$ = 0 for *i* > 10 | | $p_{i}^{ccka}$= 1 for 91 ≤ *i* ≤ 100 (new pole); $p_{i}^{ccka}$= 1 for 1 ≤ *i* ≤ 10 (old pole) and $p_{i}^{ccka}$= 0 for 10 < *i* < 91 | |
| *pleC*_H610A_ *mutant⬩* | | | | | |
| PleC | $p_{i}^{plc}$= 1 for 1 ≤ *i* ≤ 10 (old pole)  and $p_{i}^{plc}$= 0 for *i* > 10 | | | $p_{i}^{plc}$= 1 for 1 ≤ *i* ≤ 10 (old pole)  $p_{i}^{plc}$= 1 for 91 ≤ *i* ≤ 100 (new pole) and $p_{i}^{plc}$ = 0 for 10 < *i* < 91 | |
| *divK*_D90G,_ Δ*divJ*  *mutants⬩* | | | | | |
| DivL | $p_{i}^{dl}$= 1* | | | | |
| CckA | $p_{i}^{ccka}$= 1* | | | | |
| * For all *i*  ⬩ Unless specified, values of *p_i_* in the mutants are the same as that of wild type | | | | | |
