# Supplementary material for: Dynamical Localization of DivL and PleC in the Asymmetric Division Cycle of Caulobacter crescentus: A Theoretical Investigation of Alternative Models
Source: PLoS Comput Biol. 2015 Jul 17;11(7):e1004348. doi: 10.1371/journal.pcbi.1004348 (PMC4505887; doi:10.1371/journal.pcbi.1004348)
Supplement: S5 Table — (DOCX) [file pcbi.1004348.s009.docx]

**Table S5 : Summary of simulation results***

|  | Swarmer-to-stalked transition | G1-to-S transition | Predivisional cell | | | |
| --- | --- | --- | --- | --- | --- | --- |
|  |  |  | Before compartmentalization | | After compartmentalization | |
|  |  |  | PleC function | CtrA~P gradient | PleC function in swarmer compartment | DivK~P in swarmer compartment |
| WT | **+** | **+** | kinase | **+** | phosphatase | **–** |
| DivK overexpressed (4×) | **+** | **+** | kinase | **–** (☐) | phosphatase | **–** |
| DivK overexpressed (8×) | **–** | **–** (🞊) | kinase | **–** (☐) | kinase | **+** |
| DivL delocalized | **+** | **+** | kinase | **–** (☐) | phosphatase | **–** |
| DivL misclocalized | **+** | delayed | kinase | **+** | phosphatase | **–** |
| DivL and PleC mislocalized | **+** | **–** | kinase | **–** (☐) | phosphatase | **–** |
| Δ*pleC* | **–** | **+** | NA | **–** (☐) | NA | **+** |
| *pleC*_F778L_ | **+** | **+** | kinase (🞆) | **+** | phosphatase | **­–** |
| *pleC*_H610A_ | **–** | **+** | kinase (🞆) | **–** (☐) | kinase | **+** |
| *divK*_D90G_ | **–** | **–** (⬩) | phosphatase | **–** (◼) | phosphatase | **–** |
| * Cells in green are predictions of the model  🞊 Cell is in the stalked stage, accumulating chromosomes.  ⬩ G1-arrest.  🞆 PleC is in the kinase conformation but cannot catalyze phosphotransfer reaction.  ☐ CtrA is not phosphorylated in the predivisional stage.  ◼ CtrA is phosphorylated in the predivisional stage. | | | | | | |
